# FOP Negatively Regulates Ciliogenesis and Promotes Cell Cycle Re-entry by Facilitating Primary Cilia Disassembly

**DOI:** 10.3389/fcell.2020.590449

**Published:** 2020-11-12

**Authors:** Huadong Jiang, Shanshan Liu, Man-Hei Cheung, Aftab Amin, Chun Liang

**Affiliations:** ^1^State Key Lab for Molecular Neuroscience, Division of Life Science, Center for Cancer Research, Hong Kong University of Science and Technology, Hong Kong, China; ^2^Guangdong Key Laboratory for Genome Stability and Disease Prevention, Department of Pharmacology, Carson International Cancer Center, Shenzhen University Health Science Center, Shenzhen, China; ^3^Institute of Food Safety and Nutrition, Jinan University, Guangzhou, China; ^4^EnKang Pharmaceuticals (Guangzhou), Ltd., Guangzhou, China; ^5^Intelgen Limited, Hong Kong-Guangzhou-Foshan, China

**Keywords:** FOP, primary cilia, cilia assembly and disassembly, cell cycle exit and re-entry, AURKA

## Abstract

Primary cilia are microtubule-based, antenna-like organelles, which are formed in G_0_ phase and resorbed as cells re-enter the cell cycle. It has been reported that primary cilia can influence the timing of cell cycle progression. However, the molecular links between ciliogenesis and cell cycle progression are not well understood. The Fibroblast Growth Factor Receptor 1 Oncogene Partner (FOP) has been implicated in ciliogenesis, but its function in ciliogenesis is not clear. Here, we show that FOP plays a negative role in ciliogenesis. Knockdown of FOP promotes cilia elongation and suppresses cilia disassembly. In contrast, ectopic expression of FOP induces defects in primary cilia formation, which can be rescued by either pharmacological or genetic inhibition of Aurora kinase A which promotes cilia disassembly. Moreover, knockdown of FOP delays cell cycle re-entry of quiescent cells following serum re-stimulation, and this can be reversed by silencing Intraflagellar Transport 20 (IFT20), an intraflagellar transport member essential for ciliogenesis. Collectively, these results suggest that FOP negatively regulates ciliogenesis and can promote cell cycle re-entry by facilitating cilia disassembly.

## Introduction

Primary cilia are microtubule-based organelles that protrude from the cell apical surface to sense environmental cues that regulate cell growth, development, and homeostasis (Satir and Christensen, 2007; Nigg and Raff, 2009; Satir et al., 2010; Ishikawa and Marshall, 2011; Malicki and Johnson, 2017). As cell signaling centers, primary cilia coordinate with many cell signaling pathways, including those mediated by Hedgehog, Wnt, and Platelet-derived Growth Factor-α (PDGFRα) (Wallingford and Mitchell, 2011; Bangs and Anderson, 2017; Christensen et al., 2017; Malicki and Johnson, 2017; Liu et al., 2018). Defects in cilia assembly and functions lead to a wide range of human disorders, termed ciliopathies, characterized by intellectual disability, polycystic kidney, retinal defects, obesity, diabetes and other development abnormalities (Hildebrandt et al., 2011; Reiter and Leroux, 2017).

Ciliogenesis is a complex, multiple-step process occurring sequentially, and controlled by a large number of proteins, such as Rab8a, CP110, CEP290, and Intraflagellar Transport (IFT) members (Sánchez and Dynlacht, 2016; Wang and Dynlacht, 2018). While primary cilia assembly has been extensively studied, much less is known about the molecular mechanism underlying primary cilia disassembly (resorption). Recently, several cell cycle-related kinases, such as Aurora Kinase A (AURKA) and Polo-Like Kinase 1 (PLK1), have been demonstrated to play essential roles in primary cilia disassembly (Sánchez and Dynlacht, 2016). AURKA is mainly involved in mitotic events, such as centrosome duplication, separation and maturation, and spindle formation (Willems et al., 2018). However, it also exerts some non-mitotic roles in the regulation of primary cilia formation (Bertolin and Tramier, 2020). AURKA can be phosphorylated by Human Enhancer of Filamentation 1 (HEF1, also known as NEDD9). Activated AURKA in turn phosphorylates Histone Deacetylase 6 (HDAC6) and stimulates its tubulin deacetylation activity, resulting in the destabilization of the ciliary axoneme and thus cilia resorption (Pugacheva et al., 2007; Kinzel et al., 2010; Inoko et al., 2012). In addition, AURKA can be activated by Trichoplein (Inoko et al., 2012), INPP5E (phosphoinositide 5-phosphatase) (Plotnikova et al., 2015), Pitchfork (Pifo) (Kinzel et al., 2010), and calcium/calmodulin (Plotnikova et al., 2012). PLK1 can activate Kinesin Family Member 2A (KIF2A), which has microtubule-depolymerizing activity, and thus inducing cilia disassembly. PLK1 is recruited by PCM1 in G_2_ phase to the pericentriolar matrix where it interacts with and phosphorylates HDAC6 and promotes cilia deacetylation and disassembly, independently of AURKA. PLK1 also interacts with phosphorylated Disheveled 2 (Dvl2), which is mediated by both non-canonical Wnt5a signaling and Casein Kinase 1 epsilon (CK1ε), preventing HEF1, and AURKA degradation (Lee et al., 2012). These data suggest an interplay between AURKA and PLK1 mediating cilia disassembly.

The assembly and disassembly of primary cilia are tightly controlled in the cell cycle. Primary cilia are formed in quiescent (G_0_ phase) cells and are resorbed as cells re-enter the cell cycle (Sánchez and Dynlacht, 2016). Primary cilia emanate from the mother centrioles and share the same centrioles with centrosomes. When a quiescent cell enters the proliferative cycle, the centrioles need to be released from primary cilia to act as spindle poles in mitosis. It is therefore assumed that primary cilia also function as a structural checkpoint for cell cycle re-entry (Basten and Giles, 2013; Ke and Yang, 2014; Izawa et al., 2015). This hypothesis has been verified by several studies. For example, depletion of Nuclear Distribution Gene Homolog 1 (Nde1) or Dynein Light Chain Tctex-type 1 (Tctex-1) delays cell cycle re-entry in response to growth-factor stimulation by inhibiting primary cilia disassembly (Kim et al., 2011; Li et al., 2011). In contrast, silencing of proteins required for ciliogenesis, such as Intraflagellar Transport 88 (IFT88) and Kinesin Family Member 3A (KIF3A), facilitates cell proliferation (Deng et al., 2018).

The FGFR1 Oncogene Partner (FOP) was first identified as the fibroblast growth factor receptor 1 (FGFR1) oncogene fusion partner in the leukemia-associated chromosomal translocation (Popovici et al., 1999). It is a centrosomal protein, interacts with CEP350 and EB1, and promotes MT (microtubule) anchoring at the centrosome (Yan et al., 2006). The expression and subcellular localization of FOP are cell cycle regulated. In HeLa cells, it predominantly accumulates at the centrosome with low levels in G_0_ phase cells, while redistributes to centriolar satellites with increasing levels when cells enter S/G_2_/M phases (Lee and Stearns, 2013). Several studies have suggested that FOP is involved in the process of primary cilia formation; however, the functional roles of FOP in ciliogenesis are still not fully understood. Silencing of FOP in RPE1 cells did not cause primary cilia formation defects in an RNAi screen for proteins involved in ciliogenesis (Graser et al., 2007). In contrast, other studies reported that almost complete depletion or knockout of FOP severely inhibited primary cilia formation, possibly due to the impairment of the recruitment of the CEP19-RABL2 complex to the ciliary base, which allows IFT entry and initiates ciliogenesis (Lee and Stearns, 2013; Kanie et al., 2017; Mojarad et al., 2017). Apart from its roles in MT anchoring and primary cilia formation, FOP is also involved in cell cycle regulation and is upregulated in lung cancer cells (Mano et al., 2007; Acquaviva et al., 2009).

The main role of centriolar satellites is transporting proteins to the centrosome in a microtubule-based, dynein-dependent manner, and therefore centriolar satellites are regarded as the positive regulators in ciliogenesis. However, recent studies revealed that some centriolar satellite proteins also negatively regulate primary cilia formation (Prosser and Pelletier, 2020). Interestingly, a homolog of FOP, Oral-Facial-Digital syndrome 1 (OFD1), localizes to both centrioles and centriolar satellites, and exerts different effects on ciliogenesis. The centriole pool of OFD1 is essential for primary cilia formation; however, the centriolar satellites’ pool needs to be removed from centriolar satellites via autophagy at the early stage of ciliogenesis (Singla et al., 2010; Tang et al., 2013). Of note, FOP is also removed from centriolar satellites in G_0_ phase when primary cilia are formed. Moreover, the expression levels of FOP in HeLa cells are low in G_0_ phase and peak in S/G_2_ phases, which inversely correlates with ciliogenesis (Lee and Stearns, 2013). These observations suggest that FOP, apart from its implied positive role in ciliogenesis, may also act as a negative regulator in primary cilia assembly.

In this study, in contrast to previous reports (Lee and Stearns, 2013; Kanie et al., 2017; Mojarad et al., 2017), we show that FOP plays a negative role in ciliogenesis. Knockdown of FOP increases cilia length, while ectopic overexpression of FOP suppresses cilia growth in serum-starvation conditions. Moreover, during cell cycle re-entry, knockdown of FOP inhibits cilia disassembly, and overexpression of FOP accelerates cilia resorption. In addition, FOP induced cilia shortening and disassembly can be prevented by either pharmacological or genetic inhibition of AURKA. Finally, knockdown of FOP delays cell cycle re-entry of quiescent cells following serum re-stimulation, while disruption of ciliogenesis by IFT20 depletion abolishes the delay in cell cycle re-entry caused by FOP knockdown. Together, these data suggest that FOP promotes cilia shortening and disassembly via AURKA-mediated signaling, thereby linking the dynamics of ciliogenesis to cell cycle progression.

## Materials and Methods

### Cell Line and Culture

hTERT-RPE1, HEK293T, and NIH-3T3 cells were obtained from the American Type Culture Collection (ATCC). hTERT-RPE1 cells were grown in DMEM/F12 (Life Technologies) supplemented with 10% fetal bovine serum (FBS) and 0.01 mg/mL hygromycin B, hTERT-RPE1 cells stably expressing GFP or FOP-GFP were cultured as above, with the addition of G418 (0.5 mg/mL). HEK293T and NIH-3T3 cells were grown in DMEM (Life Technologies) supplemented with 10% FBS. All cells were maintained in a humidified incubator at 37°C with 5% CO_2_.

### Plasmid Construction, Stable, and Transient Transfection

GFP-tagged human FOP expression plasmid for mammalian cells was constructed by PCR and standard cloning techniques. Briefly, the human FOP (NM_007045.3) open reading frame (ORF) was amplified from human cDNA (reverse transcription products from total RNA isolated from HEK293T cells) using the following primers: FOP/Forward: 5′-CGGAATTCCGAGCAAGATGGCGGCGAC-3′, FOP/Reverse: 5′-GGGGTACCCCTGCAACATCTTCCAGATAATC-3′.

The PCR product was purified, cut with *Eco*RI and *Xho*I and then inserted into pEGFP-N1 (Clonetech). The construct was verified by DNA sequencing.

Cell transfection with plasmid DNA was performed using Lipofectamine 2000 (Invitrogen) according to the manufacturer’s instructions. RPE1 cells were plated 12–24 h before transfection. The cell confluency was about 80% at the time of transfection. For the generation of FOP-GFP stable cell lines, 48 h post-transfection, RPE1 cells were selected with 2 mg/mL G418 for approximately 2 weeks. The expression levels of FOP-GFP in individual clones were determined by immunoblotting.

Cell transfection with siRNAs was performed using Lipofectamine RNAiMAX (Invitrogen) according to the manufacturer’s instructions. Cells were seeded 12 h before transfection. The cell confluency was approximately 30% at the time of transfection. The final siRNAs concentrations were 40 nM for human FOP, mouse FOP and AURKA, and 80 nM for IFT20. For double transfection, RPE1 cells were first transfected with either the negative control siRNA or IFT20 siRNA. Cells were then split about 24 h after the first transfection. After another 24 h of culturing, cells were transfected with the negative control siRNA or FOP siRNAs for 48 h. FOP siRNAs (sense strand, #1: 5′-CCCAUUCCUAAGCCAGAGAAA-3′, #2: 5′-CG AGAGAAUUUAGCCCGAGAU-3′, #3: 5′-GGAUCACUUGGA UUAGGAA-3′, #4: 5′-GCCCGAGAUUUAGGUAUAA-3′, #5: 5′-GTGATCAGGCGCTGTCAAC-3′ (Lee and Stearns, 2013), and siFOP(3′UTR): GCAUGAUGAAAGGUGUCAAUA), mouse FOP siRNA (sense strand: 5′-GCUAGUCUCGUCGCAGAAU-3′), pooled AURKA siRNAs (sense strand, 5′-TCCCAGCGCA TTCCTTTGCAA-3′ and 5′-CAGGGCTGCCATATAACCTGA-3′) (Inoko et al., 2012; Kasahara et al., 2018), IFT20 siRNA (sense strand, 5′-GCUCGGAACUUGCUCAAAU-3′), Negative control siRNA (sense strand, 5′-UUCUCCGAACGUGUCACGU-3′). All siRNAs were purchased from GenePharma (Shanghai, China).

### Generation FOP Knockout Cells by CRISPR-Cas9 Technology

To generate FOP knockout (KO) cell lines, we applied CRISPR-Cas9 technology according to a published protocol (Ran et al., 2013). We used CHOCHOP^1^ to design a sgRNA (FOP sgRNA#1; target sequence: 5′-CGGGGTCCTGAACCGCATCA-3′), and employed another sgRNA previously described (FOP sgRNA#2; target sequence: 5′-GGGACCTGCTGGTGCAGACGCT-3′) (Mojarad et al., 2017). Both sgRNAs target the exon 1 of the *FOP* gene. The oligos were annealed and cloned into pSpCas9(BB)-2A-GFP (PX458) (Addgene: #48138, a kind gift from Feng Zhang). RPE1 cells were transfected with the PX458-FOP sgRNA constructs using FuGene 6 (Progema) according to the manufacturer’s instructions. Some 48 h after transfection, cells were trypsinized. GFP-positive cells were sorted by the BD FACSAria II Sorter (BD Bioscience), and single cells were seeded into 96-well plates. Clones were picked about 10 days later and expanded. The knockout efficacy was firstly examined by immuoblotting. Genomic DNA of the clones without FOP expression was extracted, and the sgRNA targeted locus was amplified by PCR using the following primers: Forward: 5′-GGGACCTGCTGGTGCAGACGCT-3′, Reverse: 5′-TTTATCCAGCAACAAACACGAG-3′. The PCR product was finally sequenced to confirm gene editing.

### RNA Isolation, Reverse Transcription, and qRT-PCR

Total RNA was isolated from cultured cells using TRIzol Reagent (Invitrogen), according to the manufacturer’s instructions. Reverse transcription was performed using QuantiTect Reverse Transcription Kit (QIAGEN). Real-time PCR was performed using FastStart Universal SYBR Green Master (Rox) (Roche) and LightCycler384 (Roche). The following qRT-PCR primers were used: FOP/Forward: ACAGCCAAAGTAAAGTCAAGGTT, FOP/Reverse: CACTAAACGACCGTCTTTGGTAT; AURKA/Forward: GGAATATGCACCACTTGGAACA, AURKA/Reverse: TAAGACAGGGCATTTGCCAAT; IFT20/Forward: 5′-AGCA GACCATAGAGCTGAAGG-3′, IFT20/Reverse: 5′-AGCACCG ATGGCCTGTAGT-3′; β-actin/Forward: 5′-TCCTTCCTGGGC ATGGAGTCCT-3′, β-actin/Reverse: 5′-TGCCAGGGCAGTG ATCTCCT-3′.

### Immunoblotting

Cells were harvested, washed with PBS, and lysed in RIPA buffer (150 mM NaCl, 50 mM Tris-HCl, 0.1% SDS, 1% NP-40, and 1% Triton X-100) supplemented with 1 mM PMSF (Sigma) and a protease inhibitor cocktail (Roche) at 4°C for 20 min. The lysates were then centrifuged for 15 min at 12,000 rpm at 4°C. The supernatants were collected, and an equal volume of 2X Laemmli’s buffer was added. The sample was boiled for 5 min at 95°C. Proteins were resolved by 10 or 12.5% SDS-PAGE and then transferred to nitrocellulose membranes (Pall Corporation). Membranes were blocked with 5% non-fat milk in TBST (0.1% Tween 20) for 1 h before incubation with primary and secondary antibodies sequentially. Signals were detected using SuperSignal West Pico Chemiluminescent Substrate (Thermo Fisher Scientific) according to the manufacturer’s instructions. The following antibodies were used: rabbit anti-FOP (Abcam, ab156013, 1:2,000), mouse anti-GFP (Santa Cruz, sc-9996, 1:5,000), rabbit anti-AURKA (Cell signaling Technology, 14475, 1:2,000), rabbit anti-Cyclin A2 (Abcam, ab18159, 1:10,000), rabbit anti-pCDC2 (Tyr15) (Cell Signaling Technology, 9111, 1:2,000), rabbit anti-pRb (Ser807/811) (Cell Signaling Technology, 8516, 1:2,000), and mouse anti-β-actin (Sigma, A5441, 1:5,000).

### Immunofluorescence Staining

Cells were grown on sterile glass coverslips and fixed with ice-cold methanol for 5 min at −20°C or 4% PFA for 15 min at room temperature. Cells were permeabilized with 0.5% Triton X-100 for 5 min, and blocked with 5% BSA for 1 h at room temperature, and incubated with primary antibodies overnight at 4°C and secondary antibodies 1 h at room temperature sequentially. The following primary antibodies were used: rabbit anti-γ-tubulin (Sigma, T5192, 1:1,000), mouse anti-γ-tubulin (Sigma, T5326, 1:1,000), mouse anti-acetylated tubulin (Sigma, T6793, 1: 1,000), rabbit anti-Arl13b (Proteintech, 17711-1-AP, 1:1,000), rabbit anti-FOP (Abcam, ab156013, 1:2,000), mouse anti-GFP (Roche, 11814460001, 1:1,000), rabbit anti-AURKA (Cell signaling Technology, 14475, 1:1,000) and rabbit anti-IFT88 (Proteintech, 13967-1-AP, 1:500). Secondary antibodies used were goat anti-mouse Alexa 488 (Thermo Fisher Scientific, 1:500) and goat anti-rabbit Alexa 594 (Thermo Fisher Scientific, 1:500). The nuclei were stained with 4’, 6-diamidino-2-phenylindole (DAPI) (Thermo Fisher Scientific). Slides were mounted with Prolong Gold Antifade Reagent (Thermo Fisher Scientific). Fluorescence microscopy was performed using a Nikon Elipse Ti-E or Leica DMi8 microscopy equipped with numerical aperture (NA) 1.4, oil immersion, 60 X and 100 X Plan Apo objectives. Images were acquired at room temperature using NIS-elements basis research (Nikon) or LAS X (Leica) software, and processed with Image J (NIH).

For fluorescence intensity quantification, images were taken using the same settings in the same experiment. The fluorescent intensity was measured with Image J. Briefly, the fluorescence signal (pixel area) around the basal body was selected with a tool (circle) and the integrated density (mean gray value) of the area was measured. Similarly, from the same field, an area with no fluorescence signal (next to a cell) was measured for the background fluorescence intensity. The corrected fluorescence was calculated by the formula: corrected fluorescence = integrated density − (area of the pixel with signal × mean background fluorescence intensity).

For ciliary modification assay, fluorescence intensity was measured using line-scan-based analysis in Image J, as previously described (He et al., 2014). The average intensities over a three-pixel wide line along the ciliary axoneme (marked by Arl13b) were measured and normalized against cilium length by using the ImageJ plugin Plot Roi Profile. The intensity was measured from the axonemal base to its tip in 10% intervals. The mean values of 30 cilia pooled from three independent experiments were obtained for each group.

### Fluorescence-Activated Cell Sorting (FACS) Analysis

Cells were detached with trypsin, centrifuged at 3,000 rpm for 10 min, washed twice with PBS and fixed overnight in 70% ethanol at −20°C. The cells were then resuspended and stained with PI staining buffer [5 μg/mL RNase A, 0.1% (v/v) Triton X-100, 10 mM EDTA (pH8.0) and 50 μg/mL propidium iodide (Sigma)] for 1 h at room temperature. For each sample, at least 10,000 cells were analyzed by FACSort machine (Becton Dickinson). Data were analyzed using Flowing Software 2.

### Cilia Assembly and Disassembly Assays

To induce primary cilia assembly, cells (untreated or 24 h post-transfection) were starved in serum-free medium for 48 h. In some experiments, cells were treated with DMSO (Sigma), 1 μM PHA680632 (Selleck), or 1 μM GWB43682X (Sigma), at the beginning of serum-starvation. To analyze cilia disassembly during cell cycle re-entry, cells were plated at roughly but below 30% confluency and grown overnight before transfection with siRNAs. Cells were serum-starved for 48 h immediately after transfection, and then serum was added back to the medium to 10% to stimulate cilia resorption and cell cycle re-entry. Cells were fixed at different time points and immunostained with cilia makers. The length of the cilia was measured using Image J software (NIH).

### EdU Incorporation Assay

EdU (10 μM) was added to the growth medium 2 h prior to fixation. The cells were subsequently stained according to the manufacturer’s instructions (RiboBio). Five randomly selected fields for each sample were captured using a Nikon Elipse Ti-E microscopy equipped with a numerical aperture (NA) 0.45, 20X Plan Fluor Dry objective. The numbers of the total and EdU positive cells were counted using Image J (NIH).

### Statistical Analysis

Statistical analysis was performed with Prism version 8 (GraphPad). Statistical significance of the difference between two groups was determined as indicated in the figure legend. Statistically significant differences were defined as ^∗^*p* < 0.05, ^∗∗^*p* < 0.01, ^∗∗∗^*p* < 0.001, and ^****^p < 0.0001.

## Results

### FOP Plays a Negative Role in Ciliogenesis

**FIGURE 1:**
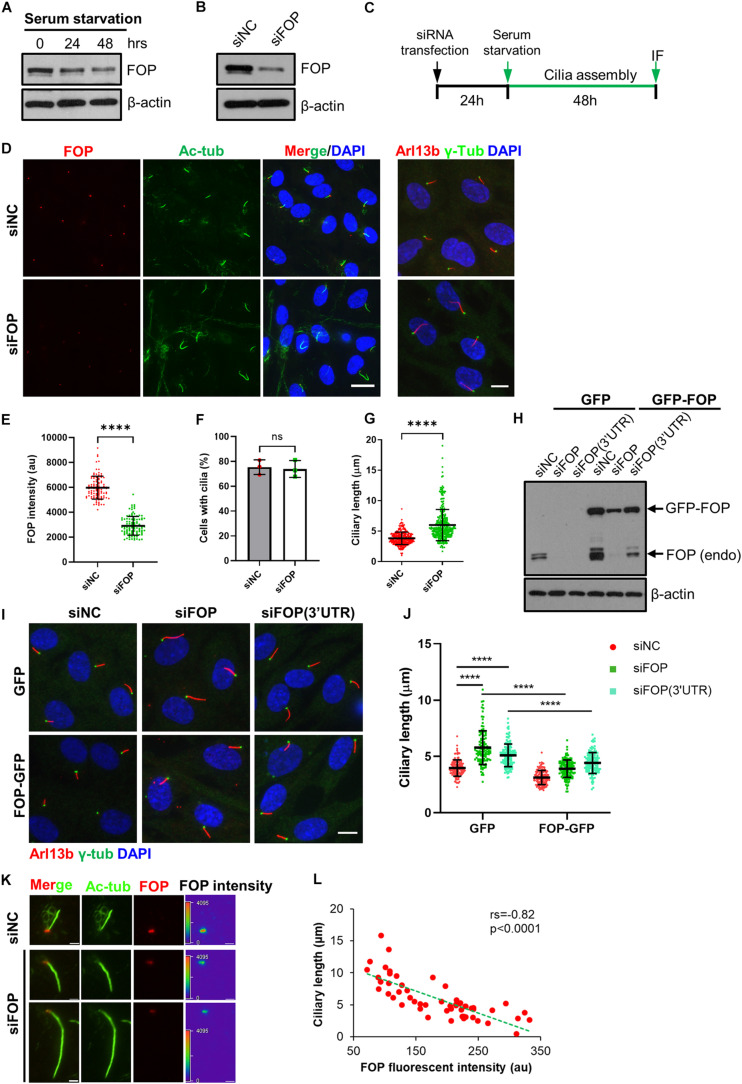
FOP negatively regulates primary cilia growth. **(A)** The protein levels of FOP decrease during cilia assembly. RPE1 cells were serum-starved to induce primary cilia formation. The levels of FOP during cilia assembly were determined by immunoblotting at 0, 24, and 48 h. **(B)** RPE1 cells were transfected with negative control siRNA (siNC) or FOP siRNAs (siFOP). The knockdown efficacy was determined by immunoblotting at 72 h post-transfection. **(C)** Schematic diagram of the experimental design for the cilia assembly assay. **(D)** RPE1 cells transfected with siNC or FOP siRNAs were serum-starved and immunostained for FOP (red) and acetylated α-tubulin (Ac-Tub; green) or Arl13b (red) and γ-tubulin (γ-Tub; green). The nuclei were stained with DAPI. Scale bars, left, 20 μm; right, 10 μm. **(E)** Quantification of FOP intensity at the ciliary base described in **(D)**; *n* = 105, 104 for siNC or siFOP treated cells, respectively; ****p < 0.0001 (unpaired, two-tailed Student *t*-test). **(F)** Quantification of the percentage of ciliated cells described in **(D)**. At least 200 cells per sample were analyzed in each experiment. **(G)** Quantification of the ciliary length in negative control cells (siNC) and FOP knockdown cells (siFOP); *n* = 327 and 325 for the negative control cells and FOP-depleted cells, respectively; ns, not significant; ****p < 0.0001 (unpaired, two-tailed, Student’s *t*-test). **(H)** RPE1 cells expressing GFP or FOP-GFP were transfected with the indicated siRNAs. The knockdown efficacy was determined by immunoblotting at 72 h post-transfection. **(I)** RPE1 cells expressing GFP or FOP-GFP were transfected with the indicated siRNAs, followed by 48 h serum starvation and immunostaining for γ-tubulin (γ-Tub; green) and Arl13b (red). The nuclei were stained with DAPI. Scale bar, 10 μm. **(J)** Quantification of the ciliary length in cells as described in **(J)**; from left to right, the cilia number *n* = 125, 119, 148, 131, 137, and 123; ****p < 0.0001 (Two-way ANOVA followed by Tukey’s multiple comparisons test). **(K)** Serum-starved RPE1 cells transfected with siNC or FOP siRNAs were immunostained for FOP (red) and acetylated α-tubulin (Ac-Tub; green). The nuclei were stained with DAPI. Scar bars, 2 μm. **(L)** The inverse relationship between the FOP intensity and ciliary length. Each red dot represents a single measurement from an individual cell (*n* = 55 cells). The green dotted line shows a linear fit through the data. Spearman correlation co-efficient (rs) and *p*-value are shown. Data are presented as mean ± SD from three independent experiments.

The assembly and disassembly of primary cilia is tightly controlled by a plethora of positive and negative regulators (Sánchez and Dynlacht, 2016; Wang and Dynlacht, 2018). Especially, proteins that negatively regulate primary cilia formation have to be degraded via ubiquitination or autophagy pathway at the early step of ciliogenesis (Tang et al., 2013; Kasahara et al., 2014). To explore the role of FOP in ciliogenesis, we first analyzed the expression of FOP during primary cilia formation. Human telomerase-immortalized retinal pigmented epithelial (hTERT-RPE1, hereafter RPE1) cells were cultured in serum-rich media or serum-starved to induce primary cilia formation. Immunoblotting analysis showed that the levels of FOP were reduced by approximately 50% during serum starvation, which is similar to the expression patterns of some negative regulators of ciliogenesis, such as CP110 and Aurora A Kinase (AURKA), in the process of primary cilia formation (Figure 1A and Supplementary Figure S1A, and data not shown). We also observed a similar reduction of ectopic FOP levels in cells stably expressing GFP-tagged FOP (FOP-GFP) in response to serum-deprivation (Supplementary Figures S1B,C). This inverse correlation between FOP expression and ciliogenesis suggests that FOP may play a negative role in ciliogenesis.

To test this hypothesis, we performed loss-of-function assays using small interfering RNA (siRNA) mediated gene silencing. RPE1 cells were transfected with a negative control siRNA (siNC) or a pool of four siRNAs (siFOP#1, #2, #3, #4) targeting FOP (hereafter siFOP). Compared to the negative control siRNA, this pool of FOP siRNA efficiently reduced the FOP protein and mRNA levels by approximately 80% (Figure 1B and Supplementary Figures S2A,B). We then investigated the effect of FOP depletion on primary cilia formation during serum starvation by immunostaining FOP and a ciliary marker, acetylated α-tubulin (Ac-Tub), or another ciliary marker, ADP-ribosylation like protein (Arl13b) and a basal body marker, γ-tubulin (γ-Tub) (Figures 1C,D). The results showed that the signals of FOP at the ciliary base were markedly decreased in FOP-depleted cells (Figures 1D,E). The percentage of ciliated cells in FOP-depleted cells was comparable to that in negative control cells (Figures 1D,F). However, the length of primary cilia was dramatically increased after FOP knockdown (Figures 1D,G), suggesting that FOP negatively regulates cilia length. These observations were confirmed by co-immunostaining acetylated α-tubulin and Intraflagellar Transport 88 (IFT88) (Supplementary Figures S2C–F). Unlike knockout (KO) of FOP, which almost completely abolishes ciliogenesis due to the failure in IFT entry (Kanie et al., 2017; Mojarad et al., 2017; Supplementary Figures S3A–E), the IFT88 signals in FOP-depleted cells appeared normal compared with those in the negative control cells (Supplementary Figure S2C), indicating that knockdown of FOP does not impair the IFT machinery.

Contrary to our results, a previous study has shown that depletion of FOP by siRNA in RPE1 cells abrogated primary cilia formation (Lee and Stearns, 2013). To rule out the possibility of off-target effects of our pooled siRNAs, we used the same siRNA (siFOP#5) employed in this previous study, and another siRNA targeting 3′-untranslated region (3′UTR) of *FOP* transcripts, siFOP(3′UTR). We transfected cells with these siRNAs (including siFOP#1, #2, #3, #4, #5, 3′UTR) individually. Immunoblotting results revealed that all of these siRNAs efficiently reduced the expression of FOP (Supplementary Figure S2G). None of them impaired cilia formation (Supplementary Figures S2H,I). On the other hand, treatment with siFOP#1, #2, #4, #5, or siFOP(3′UTR) robustly increased cilia length (Supplementary Figures S2H,J). Therefore, our results with pooled or individual siRNAs were similar. In line with knockdown of FOP in RPE1 cells, partial depletion of FOP in mouse 3T3 cells also elongated cilia, without suppressing ciliogenesis (Supplementary Figures S4A–D). These phenotypes obtained by using different siRNAs and cell lines strongly argue against off-target effects.

To further confirm the specificity of these siRNAs, we performed rescue experiments in RPE1 cells ectopically expressing GFP only or FOP-GFP that were treated with siFOP(3′UTR), which could deplete endogenous FOP, but not the ectopic FOP-GFP without 3′UTR of *FOP*. Cells expressing GFP or FOP-GFP were transfected with either siNC, siFOP or siFOP(3′UTR) followed by serum starvation to induce ciliogenesis. As expected, immunoblotting analysis showed that treatment with siFOP reduced the levels of both the endogenous FOP and ectopic FOP-GFP, but siFOP(3′UTR) only decreased the endogenous FOP (Figure 1H). After serum starvation, we found that depletion of FOP by siFOP(3’UTR) lengthened cilia in GFP expressing cells, which was mostly reversed by ectopic expression of FOP-GFP (Figures 1I,J). Although ectopic FOP-GFP was not resistant to siFOP, the residual FOP-GFP signals in FOP-GFP expressing cells transfected with siFOP were still comparable to the endogenous FOP in GFP expressing cells transfected with siNC (Figure 1H). Therefore, ectopic FOP-GFP also rescued siFOP-induced cilia elongation (Figures 1I,J). These data further excluded the possibility of off-target effects and strengthened our conclusion that FOP negatively controls cilia elongation.

Next, we investigated the relationship between the level of FOP and the length of primary cilium in individual cells. We found that the fluorescence intensity of FOP negatively correlated with the length of the corresponding primary cilia. While cells expressing high levels of FOP possessed short cilia (3–4 μm), cells expressing low levels of FOP formed longer cilia. These data support that FOP negatively regulates cilia elongation. We noted that some cells even formed extra-long cilia (>10 μm) when they still expressed low levels of FOP (Figures 1K,L). However, cells complete loss of FOP did not form cilia (Supplementary Figures S3A–E). These data suggest that a low level of FOP is required for ciliogenesis initiation, but a higher level of FOP prevents cilia lengthening.

**FIGURE 2:**
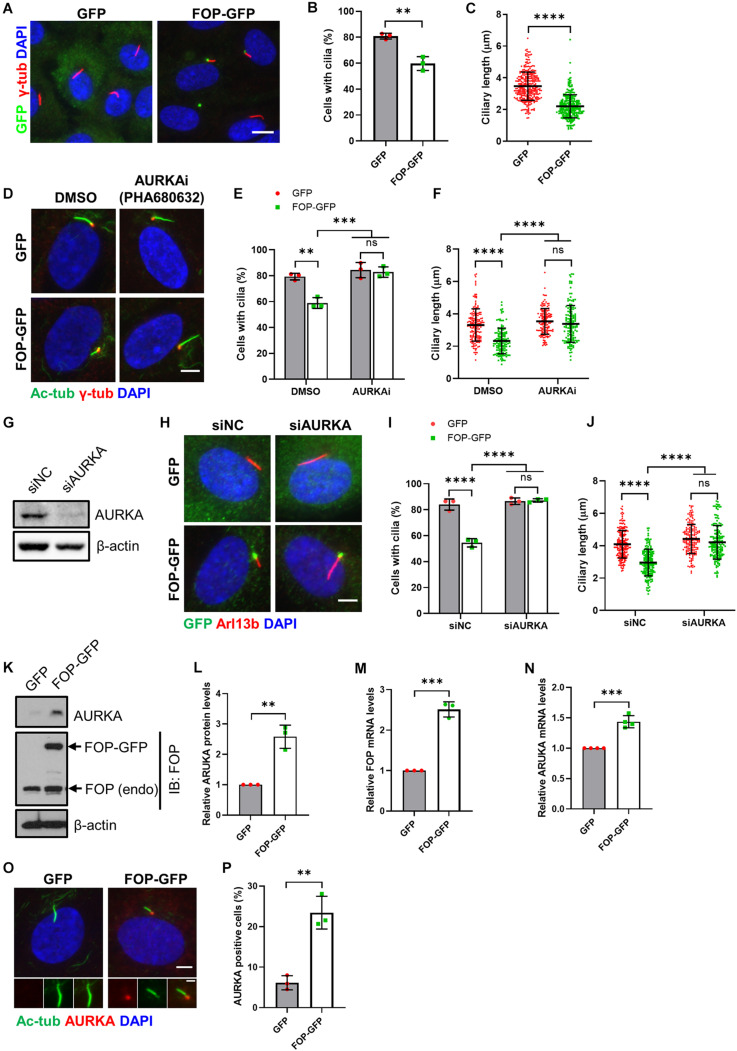
Inhibition of AURKA rescues FOP-induced ciliogenesis defects. **(A)** Serum-starved vector control cells (GFP) and FOP-overexpressed cells (FOP-GFP) were immunostained for GFP (green) and Arl13b (red). The nuclei were stained with DAPI. Scale bar, 10 μm. **(B)** Quantification of the percentage of ciliated cells. At least 200 cells per sample were analyzed in each experiment. **(C)** Quantification of the ciliary length in vector control cells and FOP-overexpressed cells; *n* = 325 and 282 for vector control cells and FOP-overexpressed cells, respectively; ns, not significant; **p < 0.01; ****p < 0.0001 (unpaired, two-tailed, Student’s *t*-test). **(D)** RPE1 cells expressing GFP or FOP-GFP were treated with DMSO or 1 μM AURKA inhibitor, PHA680632, serum-starved, and immunostained for acetylated α-tubulin (Ac-Tub; red) and γ-tubulin (γ-Tub; green). The nuclei were stained with DAPI. Scale bar, 5 μm. **(E)** Quantification of the percentage of ciliated cells described in **(D)**. At least 200 cells per sample were analyzed in each experiment; ns, not significant; **p < 0.01, ***p < 0.001 (Two-way ANOVA followed by Tukey’s multiple comparisons test). **(F)** Quantification of the ciliary length described in **(D)**. From left to right, the cilia number *n* = 170, 134, 146, and 144; ns, not significant; ****p < 0.0001 (Two-way ANOVA followed by Tukey’s multiple comparisons test). **(G)** RPE1 cells were transfected with siNC or AURKA siRNAs (siAURKA). The knockdown efficacy was determined by immunoblotting at 72 h post-transfection. **(H)** RPE1 cells expressing GFP or FOP-GFP were transfected with siNC or AURKA siRNAs, serum-starved, and immunostained for Arl13b (red) and GFP (green). The nuclei were stained with DAPI. Scale bar, 5 μm. **(I)** Quantification of the percentage of ciliated cells described in **(H)**. At least 200 cells per sample were analyzed in each experiment; ns, not significant; ****p < 0.0001 (Two-way ANOVA followed by Tukey’s multiple comparisons test). **(J)** Quantification of the ciliary length described in **(H)**. From left to right, the cilia number *n* = 197, 174, 173, and 165; ns, not significant; ****p < 0.0001 (Two-way ANOVA followed by Tukey’s multiple comparisons test). **(K)** AURKA levels in serum-starved GFP- and FOP-GFP-expressed cells. **(L)** Quantification of relative AURKA levels described in **(J)**; **p < 0.01 (unpaired, two-tailed, Student’s *t*-test). **(M,N)** qRT-PCR analysis of FOP **(M)** and AURKA **(N)** mRNA levels in GFP- and FOP-GFP-expressing cells; ***p < 0.001 (unpaired, two-tailed, Student’s *t*-test). **(O)** RPE1 cells stably expressing GFP or FOP-GFP were serum-starved and immunostained for AURKA (red) and acetylated α-tubulin (Ac-Tub; green). The nuclei were stained with DAPI. Scale bars, 5 μm (panel) and 2 μm (insert). **(P)** Quantification of the percentage of AURKA positive cells described in **(O)**. At least 100 cells per sample were analyzed in each experiment; **p < 0.01 (unpaired, two-tailed, Student’s *t*-test). Data are presented as mean ± SD from three independent experiments.

To collaborate these results obtained by the knockdown of FOP, we examined the effects of FOP overexpression on primary cilia. The results revealed that overexpression of FOP led to a decrease in ciliation, while the length of the respective cilium was severely shortened (Figures 2A–C). Taken together, the data from FOP knockdown and overexpression suggest that FOP suppresses primary cilia formation and elongation.

As primary cilia are assembled in quiescent cells when cells exit the cell cycle (Sánchez and Dynlacht, 2016), and the involvement of FOP in the regulation of cell cycle progression has been previously reported (Acquaviva et al., 2009), we therefore determined if FOP may indirectly inhibit cilia formation and elongation by preventing cell cycle exit. We examined the cell cycle profiles of FOP-depleted and FOP-overexpressed cells under serum starvation conditions by fluorescence-activated cell sorting (FACS). The cell cycle distributions of the differently treated cells were similar as most cells entered G_0_/G_1_ phases after serum starvation (Supplementary Figures S5A–D). Therefore, the inhibitory effects of FOP on primary cilia did not result from its role in cell cycle regulation.

### FOP Overexpression-Induced Ciliogenesis Defects Are Dependent on the AURKA-Mediated Cilia Disassembly Pathway

Several cell cycle-related kinases, such as AURKA and PLK1, have been demonstrated to play essential roles in primary cilia disassembly (Pugacheva et al., 2007; Inoko et al., 2012; Lee et al., 2012; Plotnikova et al., 2012, 2015; Wang et al., 2013; Miyamoto et al., 2015). AURKA is mainly involved in mitotic events, such as centrosome duplication, separation and maturation, and spindle formation (Bertolin and Tramier, 2020). In the process of cilia disassembly, it can be phosphorylated and activated by HEF1 and other proteins. Consequently, the activated AURKA further phosphorylates and activates HDAC6, which is able to deacetylate axoneme tubulin to destabilize ciliary axoneme (Pugacheva et al., 2007; Inoko et al., 2012; Plotnikova et al., 2012, 2015). Another mitotic kinase, PLK1, can phosphorylate and activate KIF2A, which in turn promotes tubulin depolymerization and thus cilia disassembly (Miyamoto et al., 2015).

To investigate the mechanism by which FOP disrupts primary cilia formation, at the beginning of serum starvation, RPE1 cells stably expressing GFP only or FOP-GFP were treated with DMSO, AURKA inhibitor PHA680632, or PLK1 inhibitor GWB43682X. Both the percentage of ciliated cells and ciliary length significantly decreased in DMSO-treated RPE1 cells stably expressing FOP-GFP compared to control cells expressing GFP only (Figures 2D–F). Pharmacological suppression of PLK1 activity did not abrogate FOP overexpression-induced ciliogenesis defects (Supplementary Figures S6A–C). On the other hand, treatment with the AURKA inhibitor restored both the percentage of ciliated cells and the length of primary cilia in RPE1 cells stably expressing FOP-GFP to the levels of the GFP expressing cells (Figures 2D–F). To further confirm these results, we used a pool of two siRNAs to efficiently silence the expression of AURKA (Figure 2G), as reported previously (Inoko et al., 2012; Kasahara et al., 2018), in RPE1 cells stably expressing GFP or FOP-GFP before serum starvation. Depletion of AURKA restored the percentage of ciliated cells and ciliary length in RPE1 cells stably expressing FOP-GFP to the levels in GFP control cells (Figures 2H–J). Together, these data strongly suggest that disruption of primary cilia formation by FOP is dependent on AURKA expression and activity.

Since inhibition of AURKA could rescue the defects in ciliogenesis induced by FOP overexpression, we questioned if FOP-overexpressed cells have higher levels of AURKA compared to vector control cells. Indeed, immunoblotting and qRT-PCR analyses revealed that the total protein and mRNA levels of AURKA were remarkably elevated in FOP-overexpressed cells under serum starvation conditions (Figures 2K–N). Immunofluorescence analysis showed that the percentage of AURKA-positive cells in FOP-overexpressed cells was significantly higher than that in GFP control cells. AURKA-positive cells were not ciliated or possessed very short cilia (Figures 2O,P). These findings suggest that FOP may stimulate AURKA expression under serum starvation conditions. This effect was not due to an increase of the population of G_2_/M cells since overexpression of FOP did not alter cell cycle distribution under serum starvation conditions (Supplementary Figures S5C,D). On the other hand, the expression levels of FOP (endogenous and exogenous) in FOP-GFP expressing cells were unaffected by AURKA depletion (Supplementary Figures S6D–F). Collectively, these data suggest that disruption of ciliogenesis by FOP overexpression is dependent on the AURKA-mediated cilia disassembly pathway, and FOP possibly induces AURKA expression.

### FOP Is Required for Timely Cilia Disassembly During Cell Cycle Re-entry

**FIGURE 3:**
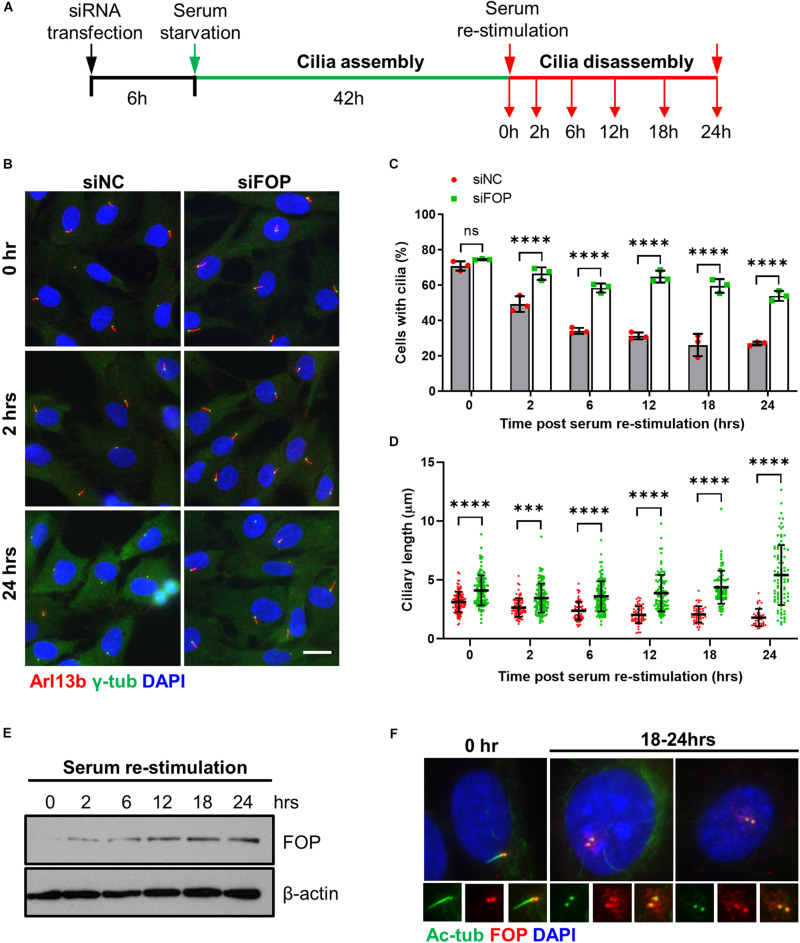
FOP is required for timely cilia disassembly. **(A)** Schematic diagram of experimental design for cilia disassembly assay. RPE1 cells transfected with siNC or FOP siRNAs were serum-starved for 48 h, followed by serum re-stimulation and fixation at different time points as indicated. **(B)** Fixed cells were immunostained for Arl13b (red) and γ-tubulin (γ-Tub; green). Representative images are shown for the 0, 2 h, and 24 h samples. The nuclei were stained with DAPI. Scale bar, 20 μm. **(C)** Quantification of the percentage of ciliated cells described in **(A)**. At least 200 cells per sample were analyzed in each experiment. **(D)** Quantification of ciliary length described in **(A)**. From left to right, the cilia number *n* = 112, 133; 75, 128; 74, 126; 54, 103; 47, 81; 38, 98. Data are presented as mean ± SD from three independent experiments; ns, not significant; ***p < 0.001; ****p < 0.0001 (Two-way ANOVA followed by Tukey’s multiple comparisons test). **(E)** Immunoblotting analysis of FOP expression during cilia disassembly. **(F)** RPE1 cells were immunostained for acetylated α-tubulin (Ac-Tub; green) and FOP (red). The nuclei were stained with DAPI. Scale bar, 20 μm.

Since FOP negatively regulates cilia formation and length, we next determined whether FOP promotes cilia disassembly when quiescent cells re-enter the cell cycle upon serum re-stimulation. After transfection with the siRNAs, the cells were serum-starved to induce cilia formation, followed by serum re-stimulated to induce cilia resorption and cell cycle re-entry (Figure 3A). Consistent with the data shown in Figures 1D–F, the percentage of ciliated cells in the negative control cells and FOP-depleted cells were similar after serum starvation, while FOP-depleted cells possessed longer cilia (0 h; Figures 3B–D and Supplementary Figures S7A–C). In response to serum re-stimulation, the primary cilia in negative control cells were resorbed in a bi-phase manner (Figures 3B–D), as described previously (Pugacheva et al., 2007). In contrast, this process was abolished in FOP-depleted cells: at all of the time points after serum addition, FOP-depleted cells had a significantly higher frequency of, and longer cilia than the negative control cells (Figures 3B–D and Supplementary Figures S7A–C). Immunoblotting analysis showed that the levels of FOP gradually increased in response to serum re-addition (Figure 3E and Supplementary Figure S7D). As cells entered S/G_2_ phases after serum re-stimulation, FOP re-localized to centriolar satellites as a previous study reported (Lee and Stearns, 2013). The primary cilia in the cells with centriolar satellites localization of FOP were short or completely resorbed (Figure 3F). These data indicate that FOP is essential for primary cilia disassembly during serum-stimulated cell cycle re-entry.

**FIGURE 4:**
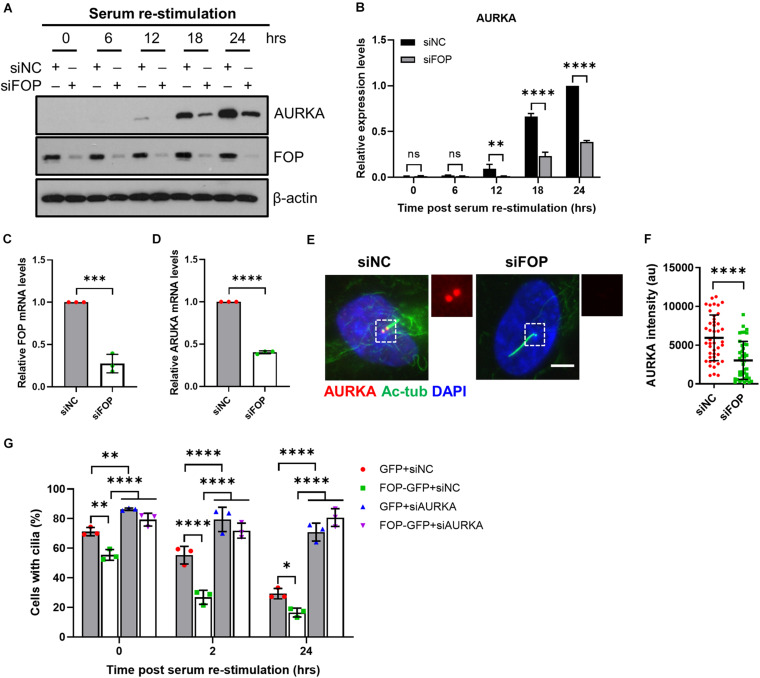
FOP-promoted cilia disassembly during cell cycle re-entry is dependent on AURKA. **(A)** Immunoblotting analysis of the total levels of AURKA in siNC or siFOP treated cells at 0, 6, 12, 18, and 24 h post serum re-stimulation. **(B)** Quantification data described in **(A)**; ns, not significant; ^∗∗^*p* < 0.01; ^****^p < 0.0001 (Two-way ANOVA followed by Tukey’s multiple comparisons test). **(C,D)** qRT-PCR analysis of FOP **(C)** and AURKA **(D)** mRNA levels in siNC and siFOP-treated cells; ^∗∗∗^*p* < 0.001, ^****^p < 0.001 (unpaired, two-tailed, Student’s *t*-test). **(E)** RPE1 cells transfected with siNC or siFOP were immunostained for AURKA (red) and acetylated α-tubulin (Ac-Tub; green). The nuclei were stained with DAPI. Scale bar, 5 μm. **(F)** Quantification of AURKA intensity at the ciliary base described in **(E)**; *n* = 45, 44 for siNC or siFOP treated cells, respectively; ^****^p < 0.0001 (unpaired, two-tailed Student *t*-test). **(G)** RPE1 cells expressing GFP or FOP-GFP were transfected with siNC or AURKA siRNAs and subjected to cilia disassembly assay. Cells were fixed at the indicated time points and immunostained for Arl13b and γ-tubulin. The percentage of ciliated cells were quantified. At least 100 cells per sample were analyzed in each experiment; ns, not significant; *p < 0.05; ^∗∗^*p* < 0.01; ^****^p < 0.0001 (Two-way ANOVA followed by Tukey’s multiple comparisons test). Data are presented as mean ± SD from three independent experiments.

Next, we further analyzed the relationship between FOP and AURKA in the process of cilia disassembly during cell cycle re-entry. We observed that the total protein and mRNA levels of AURKA during serum starvation and re-stimulation were reduced in FOP-depleted cells (Figures 4A–D). Immunofluorescence analysis also revealed that, compared to the negative control cells, the fluorescence intensity of AURKA at the ciliary base was undetectable in FOP-depleted cells, which had longer primary cilia (Figures 4E,F). While knockdown of FOP disrupted timely cilia disassembly, overexpression of FOP accelerated cilia disassembly upon serum re-stimulation (Figure 4G). Moreover, cilia disassembly in cells expressing GFP or FOP-GFP was blocked by depletion of AURKA (Figure 4G). Taken together, these data strongly suggest that FOP plays a vital role in cilia disassembly by inducing AURKA expression during cell cycle re-entry.

The aforementioned data suggest that knockdown of FOP enhances cilia stability. Post-translation modifications of axonemal microtubules, such as acetylation of lysine 40 (K40) of α-tubulin, are associated with the stability of primary cilia (Janke and Bulinski, 2011). It is possible that FOP influences cilia stability by altering post-translation modification of ciliary axoneme. To test this, we performed line-scan analysis of the fluorescence intensity. The results showed that the levels of acetylated tubulin along the ciliary axoneme (from the ciliary base to tip) were very similar in the negative control and FOP-depleted cells (Supplementary Figures S7E,F), suggesting that the effect of FOP-depletion on cilia stability may not rely on the acetylation of ciliary axoneme.

### Knockdown of FOP Delays Cell Cycle Re-entry

**FIGURE 5:**
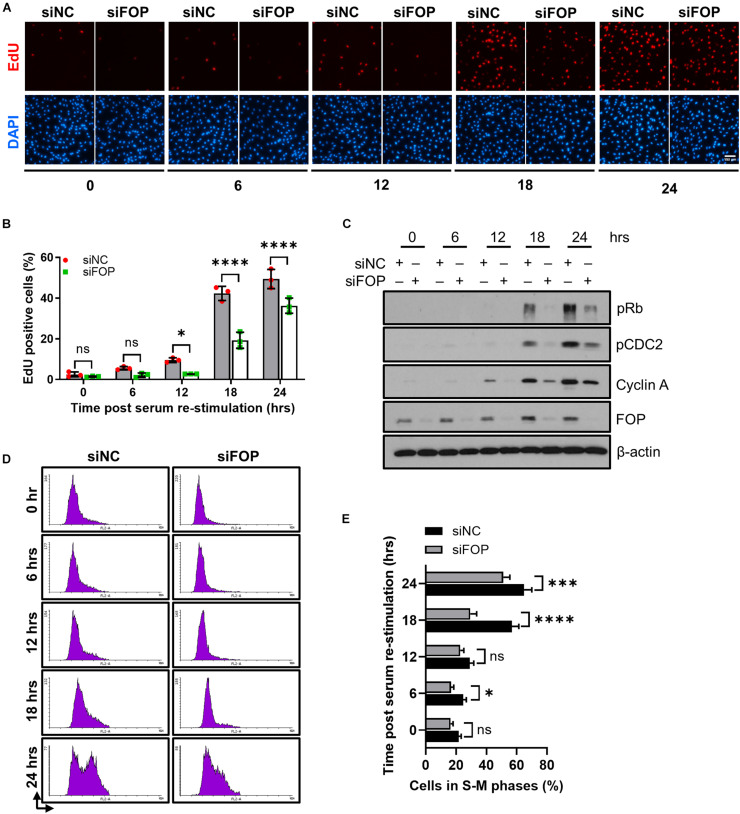
Knockdown of FOP delays cell cycle re-entry. **(A)** RPE1 cells were transfected with siNC or FOP siRNAs followed by 48 h of serum-starvation. Cells were then serum re-stimulated for the indicated durations, with cells being labeled with EdU during the final 2 h before cell harvesting. The nuclei were stained with DAPI. Scale bar, 100 μm. **(B)** Quantification of the percentage of EdU positive cells described in **(A)**. At least 500 cells per sample were analyzed in each experiment. **(C)** Immunoblotting analysis of the phosphorylation levels of Rb at Ser807/811 (pRb) and CDC2 at Tyr 15 (pCDC2) and the levels of Cyclin A and FOP in siNC and siFOP cells at 0, 6, 12, 18, and 24 h post serum re-stimulation. **(D)** Cell cycle profiles of siNC and siFOP treated cells at 0, 6, 12, 18, and 24 h after serum re-stimulation were determined by flow cytometry. At least 10,000 cells per sample were analyzed in each experiment. **(E)** Quantification of the percentage of S-M phases cells described in **(D)**. Data are presented as mean ± SD from three independent experiments; ns, not significant; *p < 0.05; ***p < 0.001; ****p < 0.0001(Two-way ANOVA followed by Tukey’s multiple comparisons test).

The disassembly of primary cilia is required for cell cycle re-entry (Kim et al., 2011; Li et al., 2011). As knockdown of FOP suppresses cilia disassembly, we therefore asked whether silencing of FOP could inhibit cell cycle re-entry. After serum starvation, cell cycle re-entry was induced by adding serum. Cells were labeled with ethynyl-deoxyuridine (EdU) after serum re-stimulation, and EdU positive cells were scored. The percentage of EdU positive cells in FOP-depleted cells was significantly lower than that of the negative control cells at 12, 18, and 24 h after serum re-stimulation (Figures 5A,B and Supplementary Figures S8A,B). Immunoblotting analysis also showed that the expression levels of cell proliferation markers, including phosphorylated Rb at Ser807/811 (pRb), phosphorylated CDC2 at Tyr15 (pCDC2) and Cyclin A, were remarkably reduced in FOP-depleted cells during cell cycle re-entry (Figure 5C and Supplementary Figures S8C–G). Correspondingly, FACS analysis also revealed a delay in cell cycle progression (Figures 5D,E). Collectively, these data indicate that knockdown of FOP delays cell cycle re-entry.

### FOP Regulates Cell Cycle Re-entry Through Modulating Primary Cilia

**FIGURE 6:**
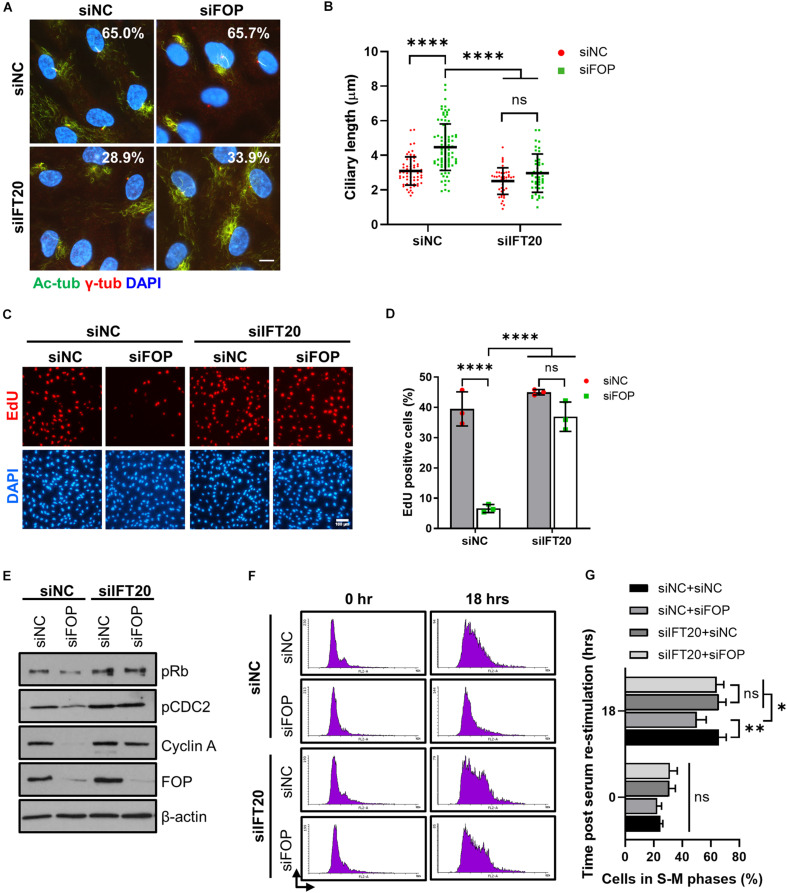
Delay in cell cycle re-entry induced by FOP knockdown is dependent of primary cilia. **(A)** RPE1 cells transfected with siNC or IFT20 siRNA were further transfected with the negative control siRNA or FOP siRNAs, serum-starved, and immunostained for γ-tubulin (γ-Tub; red) and acetylated α-tubulin (Ac-Tub; green). The nuclei were stained with DAPI. The percentage of ciliated cells from two independent experiments was indicated in the corresponding image. At least 200 cells per sample were analyzed. Scale bar, 10 μm. **(B)** Quantification of ciliary length described in **(A)**. From left to right, cilia number *n* = 64, 94, 42, and 45. **(C)** RPE1 cells were transfected with the indicated siRNAs. Following 48 h of serum-starvation, the cells were serum re-stimulated for 18 h, with cells being labeled with EdU during the final 2 h. The nuclei were stained with DAPI. Scale bar, 100 μm. **(D)** Quantification of the percentage of EdU positive cells described in **(C)**. At least 500 cells per sample were analyzed in each experiment. **(E)** Immunoblotting analysis of the levels of phosphorylated Rb at Ser807/811 (pRb), phosphorylated CDC2 at Tyr 15 (pCDC2), Cyclin A and FOP in cells transfected with the indicated siRNAs at 18 h post serum re-stimulation. **(F)** Cell cycle profiles of cells transfected with the indicated siRNAs at 0 and 18 h post serum re-stimulation were determined by flow cytometry. At least 10,000 cells per sample were analyzed in each experiment. **(G)** Quantification of the percentage of S-M phases cells described in **(F)**. Data are presented as mean ± SD from three independent experiments; ns, not significant; *p < 0.05; **p < 0.001; ****p < 0.0001 (Two-way ANOVA followed by Tukey’s multiple comparisons test).

Next, we tested if the delay in cell cycle re-entry caused by FOP knockdown is mediated by primary cilia. We silenced IFT20, a component of intraflagellar transport, to disrupt primary cilia formation (Follit et al., 2006). Consistent with previous studies, depletion of IFT20 strongly impaired cilia assembly (Supplementary Figures S9A–C). Furthermore, depletion of IFT20 dramatically decreased the ciliation in cells with or without FOP silencing, and the residual primary cilia in these cells were also obviously shortened compared to those in cells without IFT20 silencing (Figures 6A,B).

We then investigated if the loss of cilia by IFT20 silencing could overcome the delay in cell cycle re-entry induced by FOP depletion. Knockdown of FOP without ITF20 silencing (siNC, which was the control siRNA for the IFT20 siRNA) led to a significant decrease in the percentage of EdU positive cells at 18 h after serum re-stimulation, confirming the delay in cell cycle re-entry as described above. In contrast, this delay was rescued by IFT20 depletion (Figures 6C,D and Supplementary Figures S9D,E). Correspondingly, the levels of pRb, pCDC2 and Cyclin A in the FOP-silenced cells were also restored by IFT20 depletion (Figure 6E and Supplementary Figures S9F–I). FACS analysis data also showed that inhibition of ciliogenesis by IFT20 depletion rescued the delay of cell cycle progression induced by FOP knockdown (Figures 6F,G). Together, these results suggest that the delay in cell cycle re-entry induced by FOP knockdown is dependent on primary cilia.

## Discussion

FOP is a centrosomal and centriolar satellites protein and is involved in multiple biological processes, such as MT anchoring, ciliogenesis, cell cycle progression, and cancer development (Yan et al., 2006; Mano et al., 2007; Acquaviva et al., 2009; Lee and Stearns, 2013; Kanie et al., 2017; Mojarad et al., 2017; Cabaud et al., 2018). However, the roles of FOP in cilia assembly and disassembly are not fully understood. Although several recent studies have reported that near complete silencing of FOP inhibited primary cilia formation (Kanie et al., 2017; Mojarad et al., 2017; Cabaud et al., 2018), others found that moderate knockdown of FOP did not affect ciliogenesis (Graser et al., 2007; Lee and Stearns, 2013). Here we have demonstrated that FOP negatively regulates ciliogenesis based on the following evidence: firstly, the expression of FOP is inversely correlated with ciliogenesis. FOP’s levels decrease as primary cilia are formed upon serum starvation, while gradually increase as primary cilia are resorbed after serum re-addition; secondly, knockdown of FOP increases the length of primary cilia and inhibits cilia disassembly during cell cycle re-entry; thirdly, ectopic expression of FOP suppresses primary cilia formation and elongation; finally, FOP elevates the expression of AURKA, a negative regulator of ciliogenesis. The results from both the knockdown and overexpression experiments minimized the possibility of off-target effects, and confirmed that FOP indeed plays a negative role in primary cilia formation and length.

The expression of the proteins involved in ciliogenesis must be precisely regulated to exert different effects during cilia assembly and disassembly. For example, CP110 displays complex roles in ciliogenesis through the ubiquitin-proteasome system as well as transcriptional programs. High levels of CP110 suppress primary cilia formation, while optimal levels of CP110 promote ciliogenesis (Spektor et al., 2007; Kobayashi et al., 2011; Cao et al., 2012; Song et al., 2014; Walentek et al., 2016; Yadav et al., 2016). Notice that FOP does not completely degrade during ciliogenesis. Therefore, a small fraction of FOP may be necessary and sufficient for the recruitment of the CEP19-RABL2 complex to the ciliary base, allowing IFT entry and initiation of ciliogenesis. Indeed, we observed that the IFT machinery was not impaired by knockdown of FOP, possibly because the remaining FOP is sufficient for the IFT entry. As such, complete absence of FOP would compromise the essential role of FOP in the early steps of primary cilia assembly. In the present study, we uncovered a negative function of FOP in ciliogenesis. We found that FOP suppresses ciliary axoneme extension and promotes timely cilia disassembly during cell cycle re-entry, while knockdown of FOP does not impair primary cilia assembly. Although overexpression of FOP elevated the expression of AURKA and consequently led to a decrease in cilia frequency, depletion of FOP did not increase cilia number. This is possible because depletion of FOP could not further remove negative regulators or recruit positive regulators of ciliogenesis, or allow more ciliary vesicles to dock to the distal appendages. Therefore, depletion of FOP cannot enhance the initiation of ciliogenesis. After ciliogenesis initiation, FOP likely participates in the control of cilia length by modulating the balance between anterograde and retrograde IFT trains. Depletion of FOP may shift this balance to anterograde IFT trains and therefore transport more proteins to build cilia, thus increasing cilia length. Further studies are required to test these possibilities. Together with the data from others’ previous studies, our results suggest that FOP may play multiple and dose-dependent roles in ciliogenesis, being required for primary cilia formation and also playing a role in cilia disassembly. It will be interesting to elucidate how FOP’s levels are precisely controlled to produce optimal levels for ciliogenesis and cilia disassembly.

The AURKA-mediated signaling pathway is essential for cilia disassembly. AURKA has several activators including HEF1, Calmodulin and Trichoplein (Pugacheva et al., 2007; Inoko et al., 2012; Plotnikova et al., 2012, 2015). Activated AURKA further phosphorylates HDAC6, inducing cilia axoneme destabilization and disassembly. Our data suggest that FOP may serve as an upstream regulator of AURKA and induce AURKA expression and/or activation, thereby promoting cilia disassembly. Previous studies have established a link between AURKA and PLK1 in the process of ciliogenesis. PLK1 prevents HEF1 and AURKA degradation through non-canonical Wnt5a signaling, inducing cilia disassembly (Lee et al., 2012). Here, treatment with a PLK1 inhibitor, GWB43682X, did not rescue FOP-induced cilia disassembly. These data suggest FOP-induced cilia disassembly is independent of PLK1-mediated cilia disassembly signaling.

The link between ciliogenesis and the cell cycle has been established (Pugacheva et al., 2007; Kim et al., 2011; Li et al., 2011). Primary cilia have to be completely disassembled prior to mitosis, releasing the centrioles to form the mitotic spindle poles. Therefore, the length of primary cilia regulates the progression of the cell cycle. For example, depletion of Nde1 or Tctex-1 delays cell cycle progression by suppressing timely cilia disassembly (Kim et al., 2011; Li et al., 2011). The role of FOP in cell cycle progression has previously been implicated (Acquaviva et al., 2009). The data we present here suggest that FOP, like Nde1 and Tctex-1, also facilitates cell cycle re-entry by promoting cilia disassembly. Of note, our FACS data suggested that FOP does not prevent cell cycle exit. Therefore, FOP may only affect cell cycle re-entry. Given that FOP is a centrosomal protein, and loss of centrosome integrity causes G_1_-S arrest (Yan et al., 2006; Mikule et al., 2007), it could be argued that the cell cycle delay in FOP knockdown cells originated from a loss of centrosome integrity. However, as the cell cycle re-entry delay induced by FOP knockdown can be rescued by the depletion of IFT20 (which is essential for cilia assembly but not centrosome integrity; Follit et al., 2006), a possible loss of centrosome integrity cannot be the reason for cell cycle re-entry delay induced by FOP knockdown. Furthermore, in chicken DT40 lymphocytes, FOP knockout did not impair centrosome integrity (Acquaviva et al., 2009). By immunostaining γ-tubulin, we also did not detect any apparent centrosome defects during the cell cycle re-entry. Therefore, the delay in cell cycle re-entry induced by FOP knockdown is most likely mediated by primary cilia. Our data support the hypothesis that primary cilia serve as a structural checkpoint of the cell cycle.

Recent studies have suggested that primary cilia are involved in tumorigenesis and tumor progression, as the loss of cilia is frequently observed in various types of cancer such as breast cancer, prostate cancer and pancreatic ductal adenocarcinoma (Seeley et al., 2009; Yuan et al., 2010; Hassounah et al., 2013; Menzl et al., 2014). Although the mechanism is still unclear, the loss of cilia probably provides a growth advantage and promotes malignant transformation during the early stages of tumor development (Basten and Giles, 2013; Deng et al., 2018; Zingg et al., 2018). Upregulation of FOP has been observed in lung cancer tissues and cell lines (Mano et al., 2007). Here, we demonstrate that FOP promotes cilia disassembly and accelerates cell cycle progression. It is likely that upregulation of FOP induces cilia loss and provides growth advantages for cancer cells.

FOP is homologous to FOR20 (FOP-related protein of 20 kDa) and OFD1, and they have a very conserved LisH domain (Yan et al., 2006; Sedjaï et al., 2010; Singla et al., 2010). Similar to FOP, FOR20, and OFD1 also localize to both centrioles and centriolar satellites and play important roles in ciliogenesis (Sedjaï et al., 2010; Singla et al., 2010; Aubusson-Fleury et al., 2012; Tang et al., 2013). Knockdown of FOR20 by siRNA-mediated gene silencing in RPE1 cells compromises primary cilia assembly and elongation, and FOR20 knockout zebrafish mutants display many ciliopathies-related phenotypes during development (Sedjaï et al., 2010; Xie et al., 2019). OFD1 has more complex roles in ciliogenesis. The centriole pool of OFD1 concentrates at the distal end of the centriole, and it is necessary for distal appendage assembly and IFT recruitment. Therefore, this pool of OFD1 is essential for primary cilia formation; knockout of OFD1 impairs ciliogenesis. On the contrary, partial depletion of OFD1 in non-transformed RPE1 and MEF cells as well as MCF7 cancer cells using RNAi technique to remove the centriolar satellite pool while maintaining the centriole pool of OFD1 promotes primary cilia formation. Actually, OFD1 is degraded from centriolar satellites via autophagy during cilia assembly (Singla et al., 2010; Tang et al., 2013). Interestingly, the centriolar satellite pool of FOP also disappears during primary cilia assembly but reappears as primary cilia start to resorb. It is therefore possible that the centriolar satellites pool of FOP also functions as a suppressor of ciliogenesis.

Our results and data from others’ previous studies suggest that FOP plays complex roles in cilia assembly and disassembly. On one hand, the centriole pool of FOP is essential for initiating ciliogenesis by recruiting the IFT machinery. On the other hand, FOP promotes cilia shortening and disassembly by elevating the expression of AURKA, and thus facilitating cell cycle re-entry. Future studies are required to investigate how FOP regulates AURKA expression. It is also very meaningful to explore how FOP’s levels are precisely controlled to produce optimal levels for cilia assembly and disassembly, and the role of centriolar satellites pool of FOP in ciliogenesis.

## Data Availability Statement

The original contributions presented in the study are included in the article/Supplementary Material, further inquiries can be directed to the corresponding author.

## Author Contributions

HJ designed the experiments, conducted the major experiments, analyzed the data, and prepared the manuscript. SL conducted the experiments and provided reagents and materials. M-HC and AA revised the manuscript. CL conceived the project, designed and supervised the experiments, and revised the manuscript. All authors contributed to the article and approved the submitted version.

## Conflict of Interest

CL is associated with EnKang Pharmaceuticals (Guangzhou) Ltd. and Intelgen Limited. The remaining authors declare that the research was conducted in the absence of any commercial or financial relationships that could be construed as a potential conflict of interest.

## Footnotes

1 https://chopchop.cbu.uib.no
