# Supplementary material for: FOP Negatively Regulates Ciliogenesis and Promotes Cell Cycle Re-entry by Facilitating Primary Cilia Disassembly
Source: Front Cell Dev Biol. 2020 Nov 12;8:590449. doi: 10.3389/fcell.2020.590449 (PMC7693466; doi:10.3389/fcell.2020.590449)
Supplement: Supplementary file 1 [file Data_Sheet_1.pdf]

Additional files

Figure S1.

(**A**) Quantification of the relative FOP protein levels during cilia assembly described in Figure 1A; ****p<0.0001 (One-way ANOVA). (**B**) RPE1 cells stably expressing GFP or FOP-GFP were cultured in the serum presence (S+) or absence (S-) media for 48 hrs. The cell lysates were then subjected to immunoblotting for the indicated antibodies. β-actin was used as the loading control. (**C**) Quantification of the relative FOP-GFP levels (immunoblotted by the GFP antibody) described in (**B**); **p<0.01 (unpaired, two-tailed Students' t-test). Data are presented as mean ± SD from three independent experiments.

Figure S2.

(**A**) Quantification of the relative FOP protein levels described in Figure 1B. **p<0.01 (unpaired, two-tailed Students' t-test). (**B**) Quantification of the relative FOP mRNA levels of RPE1 cells transfected with the siNC or FOP siRNAs. The mRNA levels were determined by qRT-PCR; **p<0.01 (unpaired, two-tailed Students' t-test). (**C**) RPE1 cells transfected with siNC or FOP siRNAs were immunostained for acetylated α-tubulin (Ac-Tub; green) and IFT88 (red). The nuclei were stained with DAPI. Scale bars, 20 μm (panel) and 5 μm (insert). Inserts show magnification of the indicated cilia. (**D**, **E**) Quantification of cilia labeled by acetylated α-tubulin (**D**) or IFT88 (**E**) antibody. At least 200 cells per sample in each experiment were examined. (**F**) Quantification of the length of acetylated α-tubulin labeled cilia described in (**A**); n=395 or 377 for siNC or siFOP treated cells, respectively; ****p<0.01 (unpaired, two-tailed Students' t-test). (**G**)RPE1 cells were transfected with siNC or individual FOP siRNAs. The knockdown efficacy was determined by immunoblotting at 72 hrs post-transfection. (**H**) RPE1 cells transfected with siNC or individual FOP siRNAs were serum-starved and immunostained for FOP (red) and acetylated α-tubulin (Ac-Tub; green). The nuclei were stained with DAPI. Scale bars, 10 μm. (**I**) Quantification of the percentage of ciliated cells described in (**D**). At least 150 cells per sample were analyzed in each experiment. (**J**) Quantification of the ciliary length in negative control cells and FOP knockdown cells; from left to right, the cilia number n=175, 136, 154, 118, 141, 146 and 144; ns, not significant; ****p<0.0001 (One-way ANOVA). Data are presented as mean ± SD from at least three independent experiments.

Figure S3.

(**A**) Immunoblotting analysis of FOP protein levels in wild-type (WT) and two FOP-knockout (KO) cell lines, clone #1-9 and #2-10. (**B**) DNA sequencing analysis of the sgRNA targeted *FOP* gene locus in wild-type (WT) and two FOP knockout clones. (**C**) DNA sequencing analysis revealed both alleles of *FOP* in clone #1-9 and #2-10 had a 1-bp insertion and a 10-bp deletion, respectively. (**D**) Serum-starved WT and FOP-KO RPE1 cells were immunostained for γ-tubulin (γ-Tub; green) and Arl13b (red). The nuclei were stained with DAPI. Scale bars, 10 μm. (**E**) Quantification of the percentage of ciliated cells. About 100 cells per sample were analyzed in each experiment. Data are presented as mean ± SD from two independent experiments.

Figure S4.

(**A**) 3T3 cells were transfected with siNC or mouse FOP siRNA. The knockdown efficacy was determined by immunoblotting at 72 hrs post-transfection. (**B**) 3T3 cells transfected with siNC or mouse FOP siRNA were serum-starved and immunostained for γ-tubulin (γ-Tub; red) and acetylated α-tubulin (Ac-Tub; green). The nuclei were stained with DAPI. Scale bar, 20 μm. (**C**) Quantification of the percentage of ciliated cells described in (**B**). At least 150 cells per sample were analyzed. (**D**) Quantification of the ciliary length in the negative control and FOP knockdown 3T3 cells; the cilia number n=165 and 160 for the negative control and FOP knockdown 3T3 cells, respectively. Data are presented as mean or mean ± SD from one experiment; ****p<0.0001 (unpaired, two-tailed student test).

Figure S5.
(**A**) Cell cycle profiles of serum-starved (48 hrs) RPE1 cells transfected with siNC or FOP siRNAs. (**B**) Quantification of the data in (**A**). (**C**) Cell cycle profiles of serum-starved (48 hrs) RPE1 cells stably expressing GFP or FOP-GFP. (**D**) Quantification of the data in (**C**). Cell cycle profiles were analyzed by flow cytometry. Data are presented as mean ± SD from three independent experiments.

Figure S6.
(**A**) RPE1 cells expressing GFP or FOP-GFP were treated with DMSO or 1 μM PLK1 inhibitor, GW843682X, serum-starved, and immunostained for acetylated α-tubulin (Ac-Tub; red) and γ-tubulin (γ-Tub; green). The nuclei were stained with DAPI. Scale bar, 5 μm. (**B**) Quantification of the percentage of ciliated cells described in (**A**). At least 200 cells per sample in each experiment; ns, not significant; ***p<0.001 (Two-way ANOVA followed by Tukey's multiple comparisons test). (**C**) Quantification of the ciliary length described in (**A**). From left to right, the cilia number n=100, 100, 89 and 74. The DMSO treated cells were the same samples as described in Figure 2A-C; ns, not significant; ****p<0.0001 (Two-way ANOVA followed by Tukey's multiple comparisons test). (**D**) RPE1 cells stably expressing FOP-GFP were transfected with the negative control siRNA or AURKA siRNAs, serum-starved, and subjected to immunoblotting for the indicated antibodies. (**E**) The percentage of GFP positive cells in RPE1 cells stably expressing FOP-GFP treated as described in Figure 2E. At least 100 cells per sample in each experiment were examined. (**F**) GFP intensity in RPE1 cells stably expressing FOP-GFP treated as described in Figure 2E; n=33 or 40 for siNC or siAURKA treated cells, respectively; ns, not significant (unpaired, two-tailed Students' t-test). Data are presented as mean ± SD from three independent experiments.

Figure S7.
(**A**)RPE1 cells transfected with siNC or siFOP(3'UTR) were serum-starved for 48 hrs, followed by serum re-stimulation, fixed at 0 and 18 hr (s) post serum re-stimulation, immunostained for acetylated α-tubulin (Ac-Tub) and γ-tubulin (γ-Tub). The nuclei were stained with DAPI. Scale bar, 20 μm. (**B**) Quantification of the percentage of ciliated cells described in (**A**). At least 200 cells per sample were analyzed in each experiment. (**C**) Quantification of ciliary length described in (**A**). From left to right, the cilia number n=91, 105, 49 and 102. Data are presented as mean ± SD from three independent experiments; ns, not significant; ****p<0.0001 (Two-way ANOVA followed by Tukey's multiple comparisons test). (**D**) Quantification of the relative

FOP expression during cilia disassembly; **p<0.0001 (One-way ANOVA test). (**E**) RPE1 cells transfected with siNC or FOP siRNA were serum-starved and immunostained for acetylated α-tubulin (Ac-Tub; green) and Arl13b (red) to mark axonemal modification and cilia, respectively. The nuclei were stained with DAPI. Scale bar, 5 μm. (**F**) Quantification results of levels of acetylated α-tubulin, 30 cilia per samples were analyzed. Data are presented as means ± SD from three independent experiments

Figure S8.

(**A**) RPE1 cells were transfected with siNC or siFOP(3'UTR) followed by 48 hrs of serum-starvation. Cells were then serum re-stimulated for 18 hrs, with cells being labeled with EdU during the final 2 hrs before cell harvesting. The nuclei were stained with DAPI. Scale bar, 100 μm. (**B**) Quantification of the percentage of EdU positive cells described in (**A**). At least 500 cells per sample were analyzed in each experiment. (**C-F**) Quantification of the relative FOP (**C**), Cyclin A (**D**), pRb (**E**) and pCDC2 (**F**) levels during cell cycle re-entry. Data are presented as mean ± SD from three independent experiments; ns, not significant; **p<0.01; ****p<0.0001 (Two-way ANOVA followed by Tukey's multiple comparisons test). (**G**) Immunoblotting analysis of the levels of pRb, pCDC2, Cyclin A and FOP in siNC and siFOP(3'UTR)-treated cells at 18 hrs post serum re-stimulation.

Figure S9.

(**A**) The relative mRNA levels of cells transfected with the control siRNA or IFT20 siRNA were measured by qRT-PCR at 96 hrs post-transfection. (**B**) Cells transfected with the control siRNA or IFT20 siRNA were serum-starved for 48 hrs post-transfection, followed by immunostaining for acetylated-α-tubulin (green) and γ-tubulin (red). The nuclei were stained with DAPI. Scale bar, 10 μm. (**C**) Quantification of the percentage of cells with cilia described in (**B**). At least 200 cells per sample in each experiment were examined. (**D**) RPE1 cells were transfected with the indicated siRNAs. Following 48 hrs of serum-starvation, the cells were serum re-stimulated for 18 hrs, with cells being labeled with EdU during the final 2 hrs. The nuclei were stained with DAPI. Scale bar, 100 μm. (**E**) Quantification of the percentage of EdU positive cells described in (**D**). At least 500 cells per sample were analyzed in each experiment; ns, not significant; *p<0.05; **p<0.001; ****p<0.0001 (Two-way ANOVA followed by Tukey's multiple comparisons test). (**F-I**) Quantification of the relative FOP (**F**), Cyclin A (**G**), pRb (**H**) and pCDC2 (**I**) levels during cell cycle re-entry; ns, not significant; *p<0.05, **p<0.01; ***p<0.001; ****p<0.0001 (Two-way ANOVA followed by Tukey's multiple comparisons test). Data are presented as mean ± SD from three independent experiments.
