# Supplementary figures and images for: FOP Negatively Regulates Ciliogenesis and Promotes Cell Cycle Re-entry by Facilitating Primary Cilia Disassembly
Source: Front Cell Dev Biol. 2020 Nov 12;8:590449. doi: 10.3389/fcell.2020.590449 (PMC7693466; doi:10.3389/fcell.2020.590449)

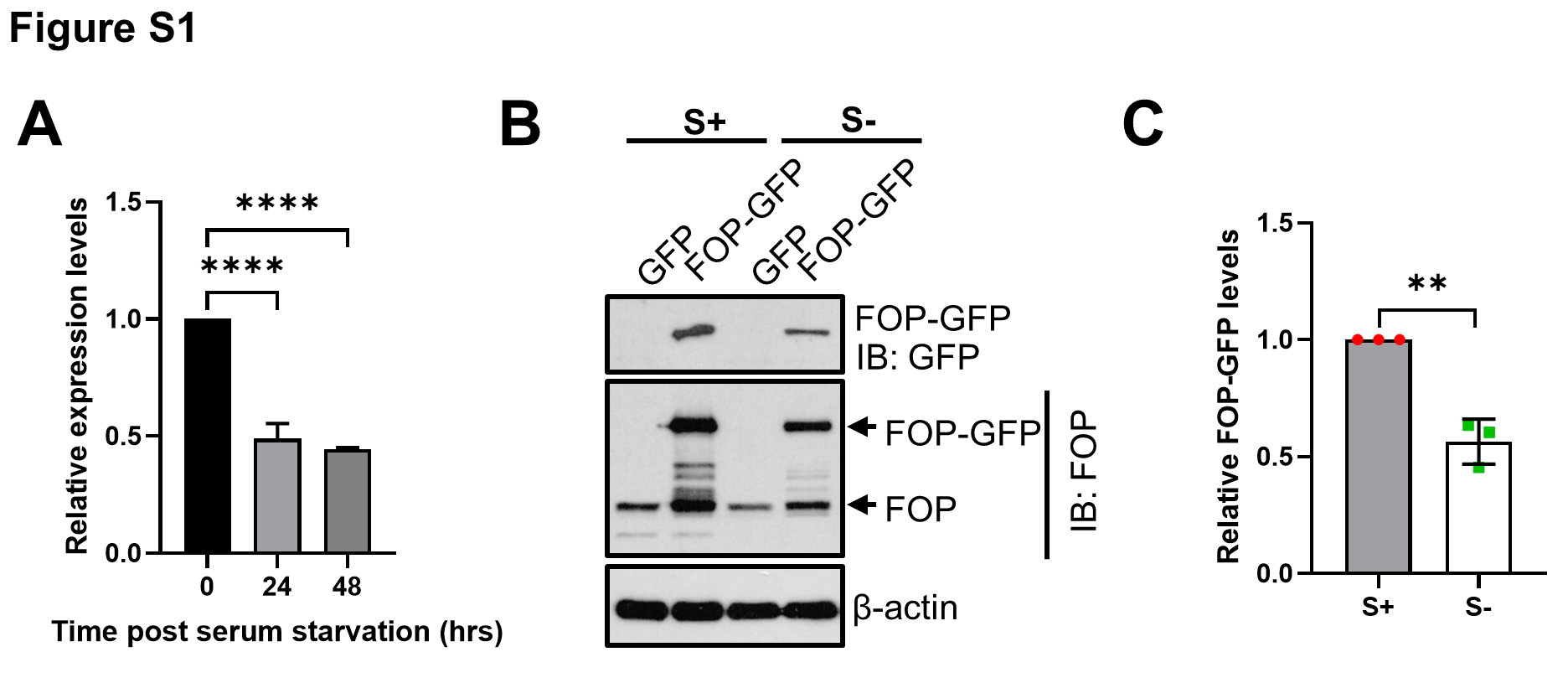

Supplement: Supplementary file 2 [file Image_1.TIF]

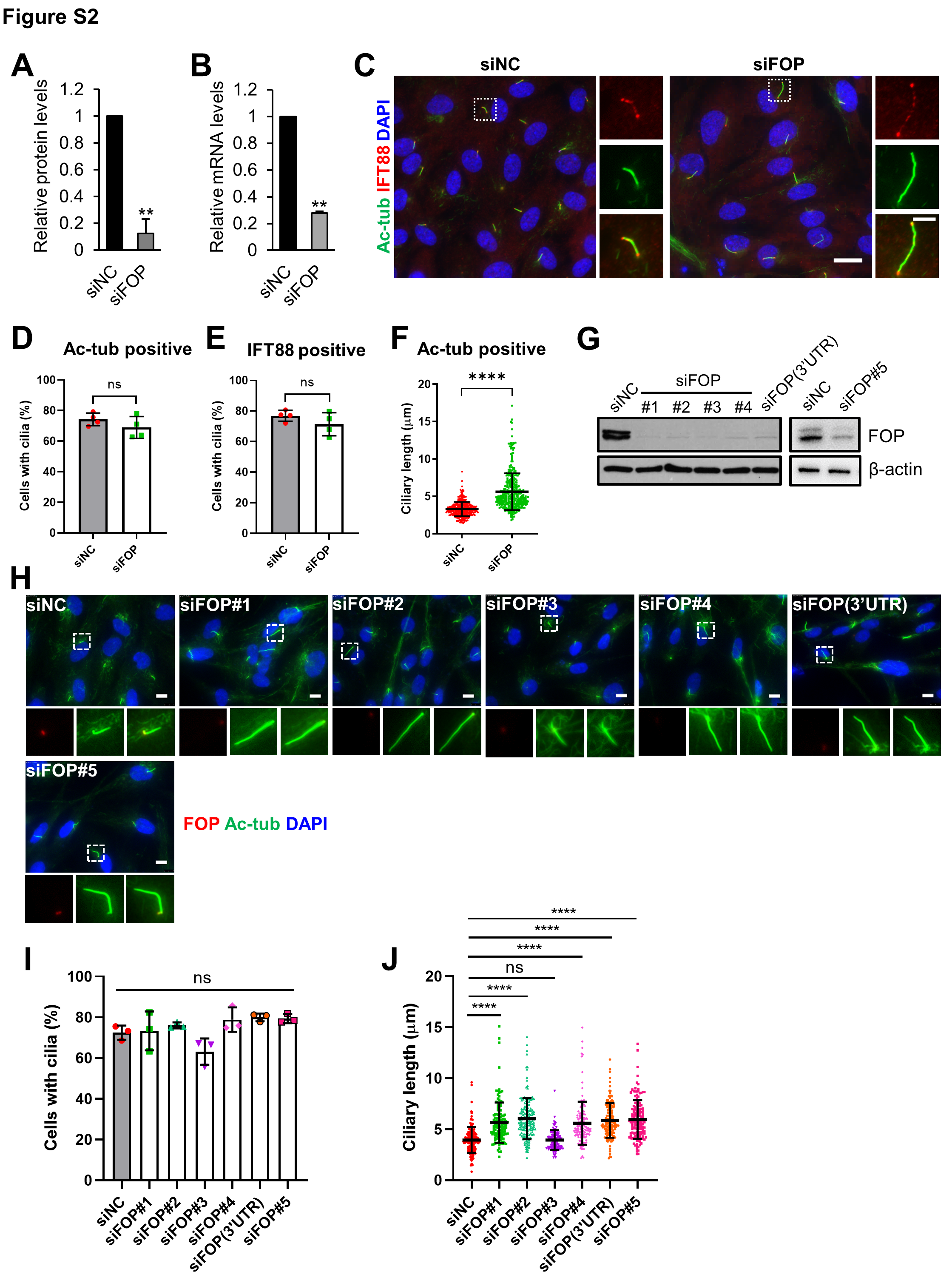

Supplement: Supplementary file 3 [file Image_2.TIF]

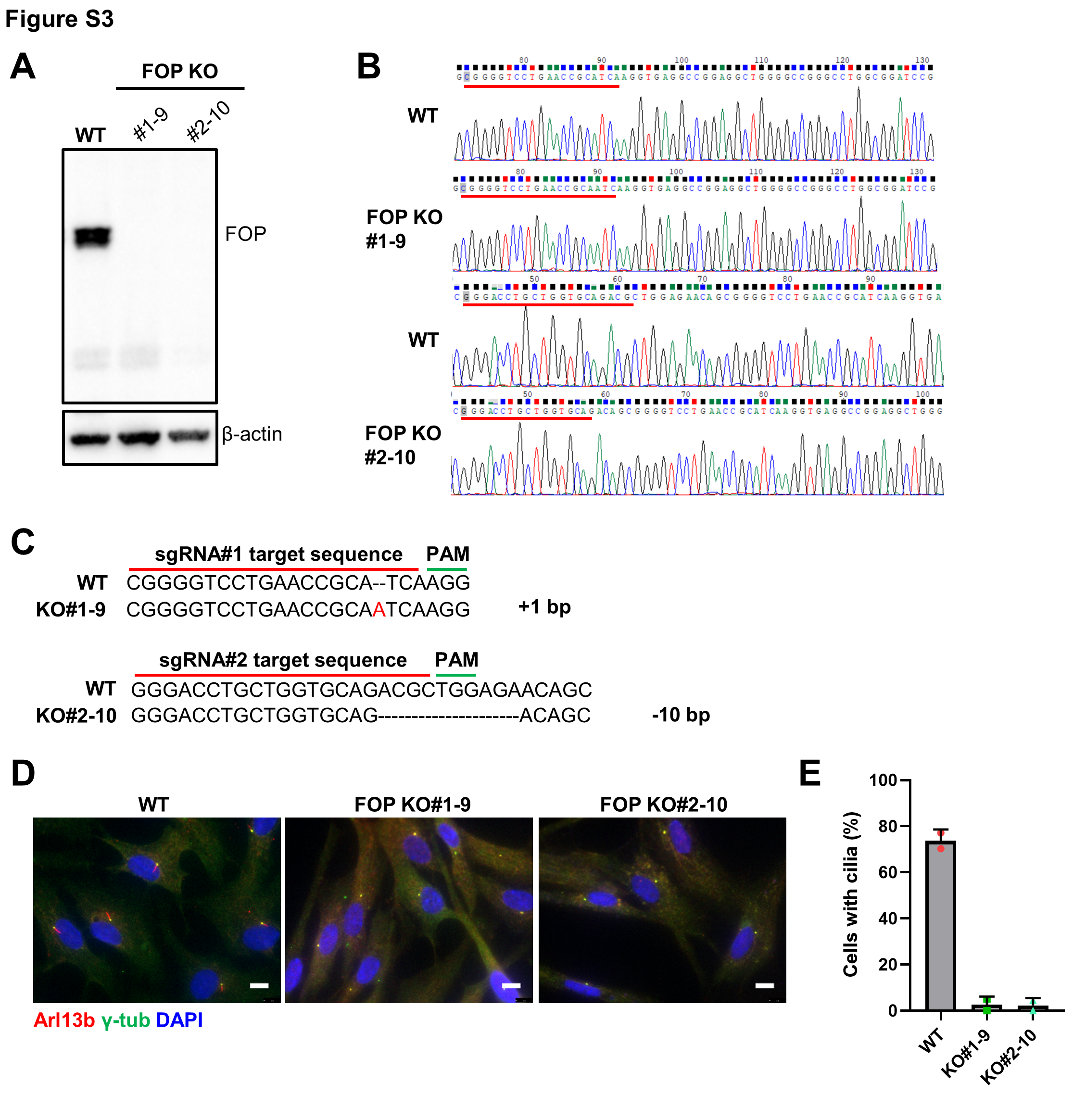

Supplement: Supplementary file 4 [file Image_3.TIF]

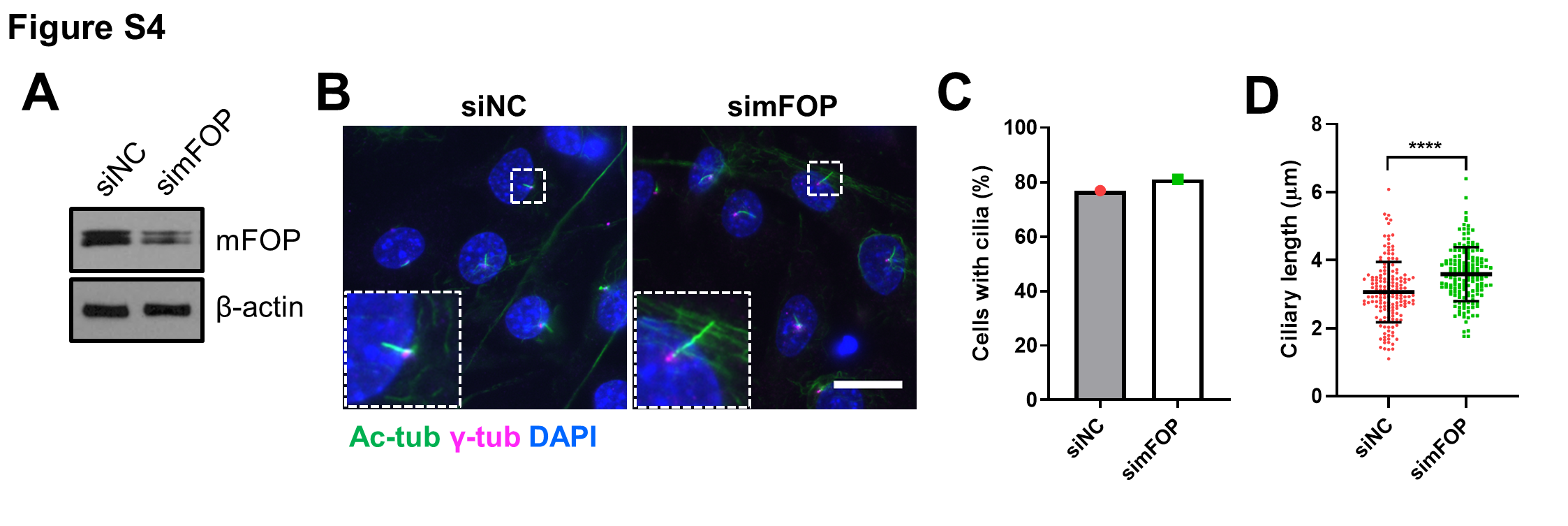

Supplement: Supplementary file 5 [file Image_4.TIF]

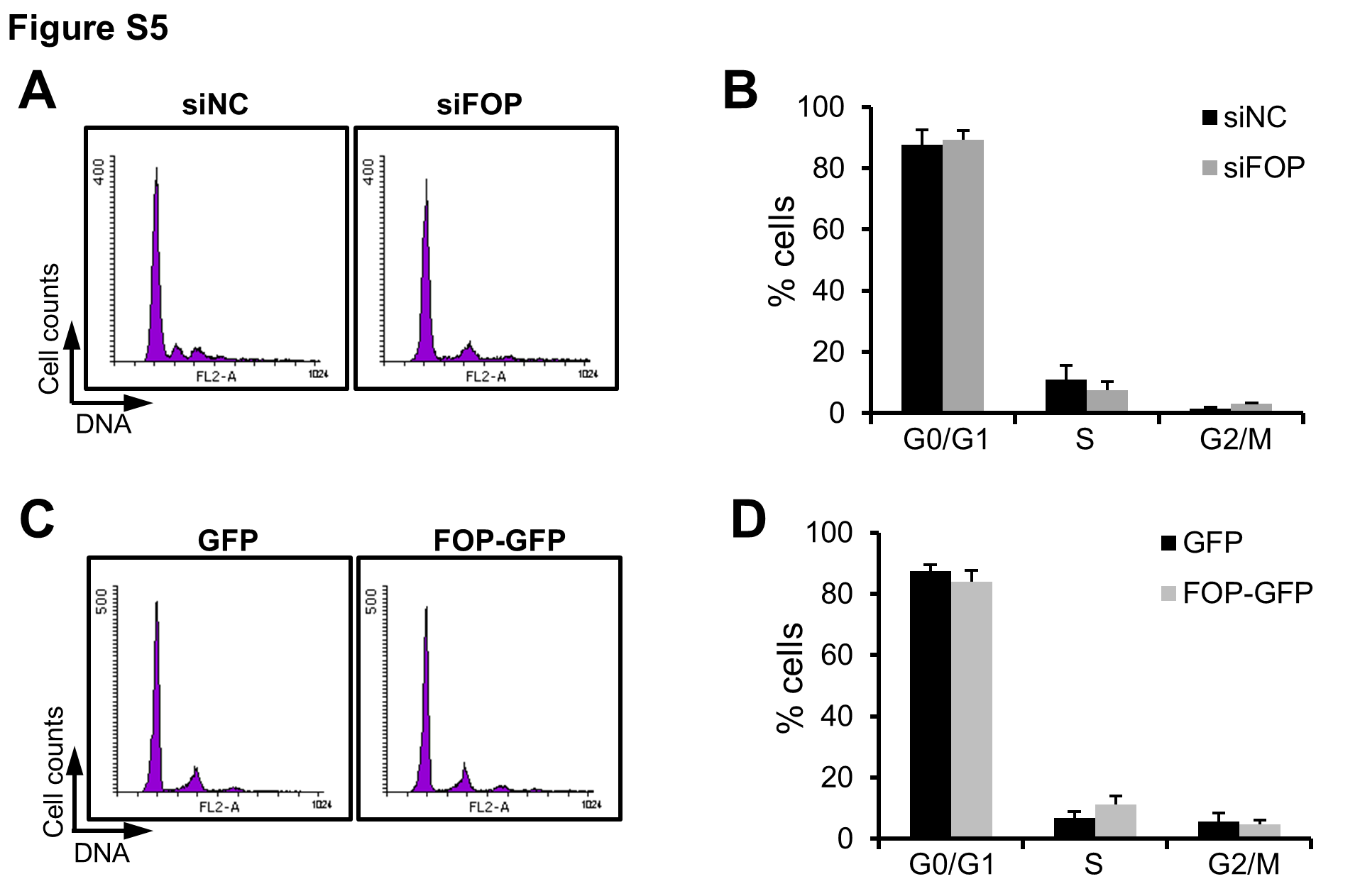

Supplement: Supplementary file 6 [file Image_5.TIF]

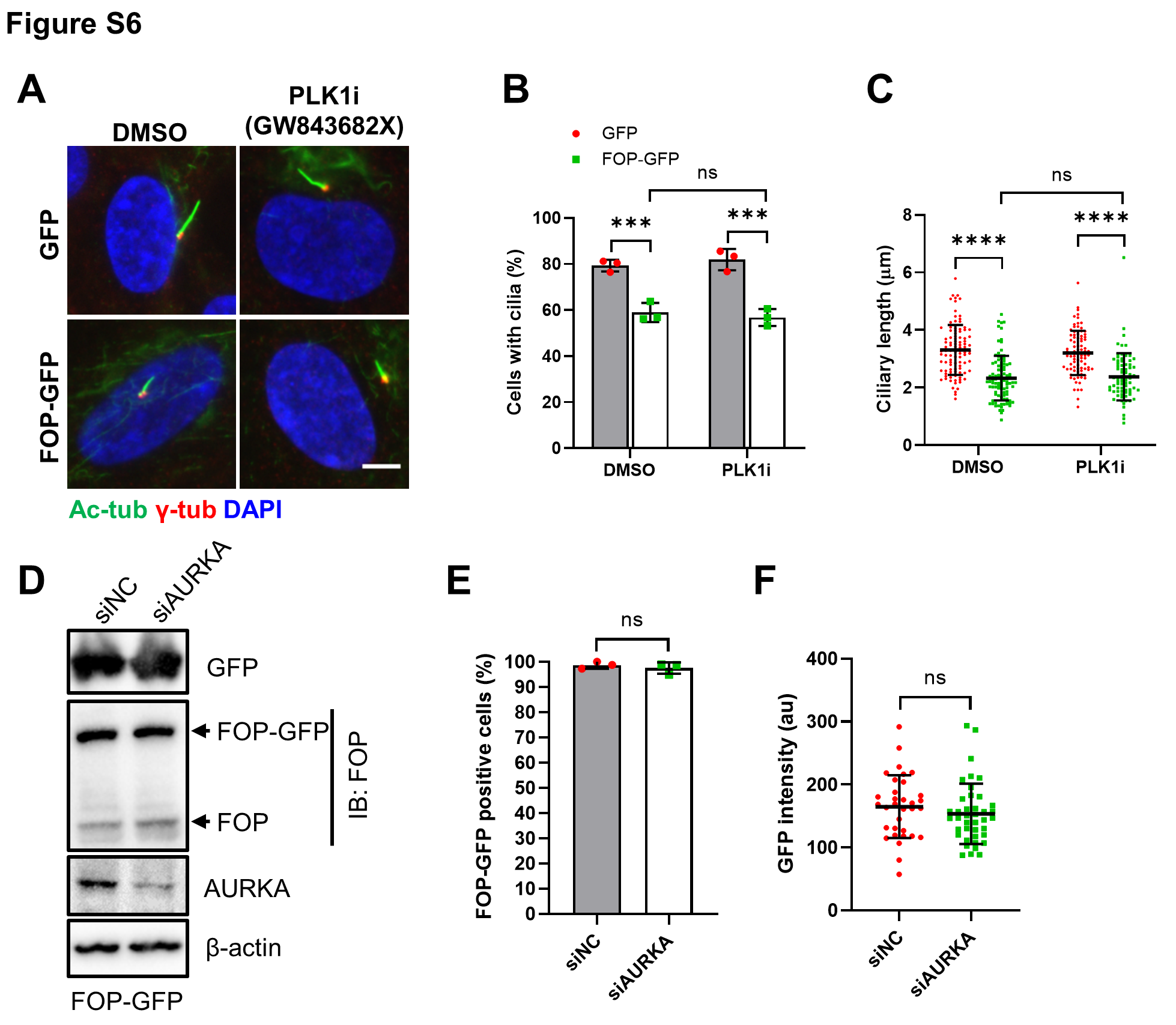

Supplement: Supplementary file 7 [file Image_6.TIF]

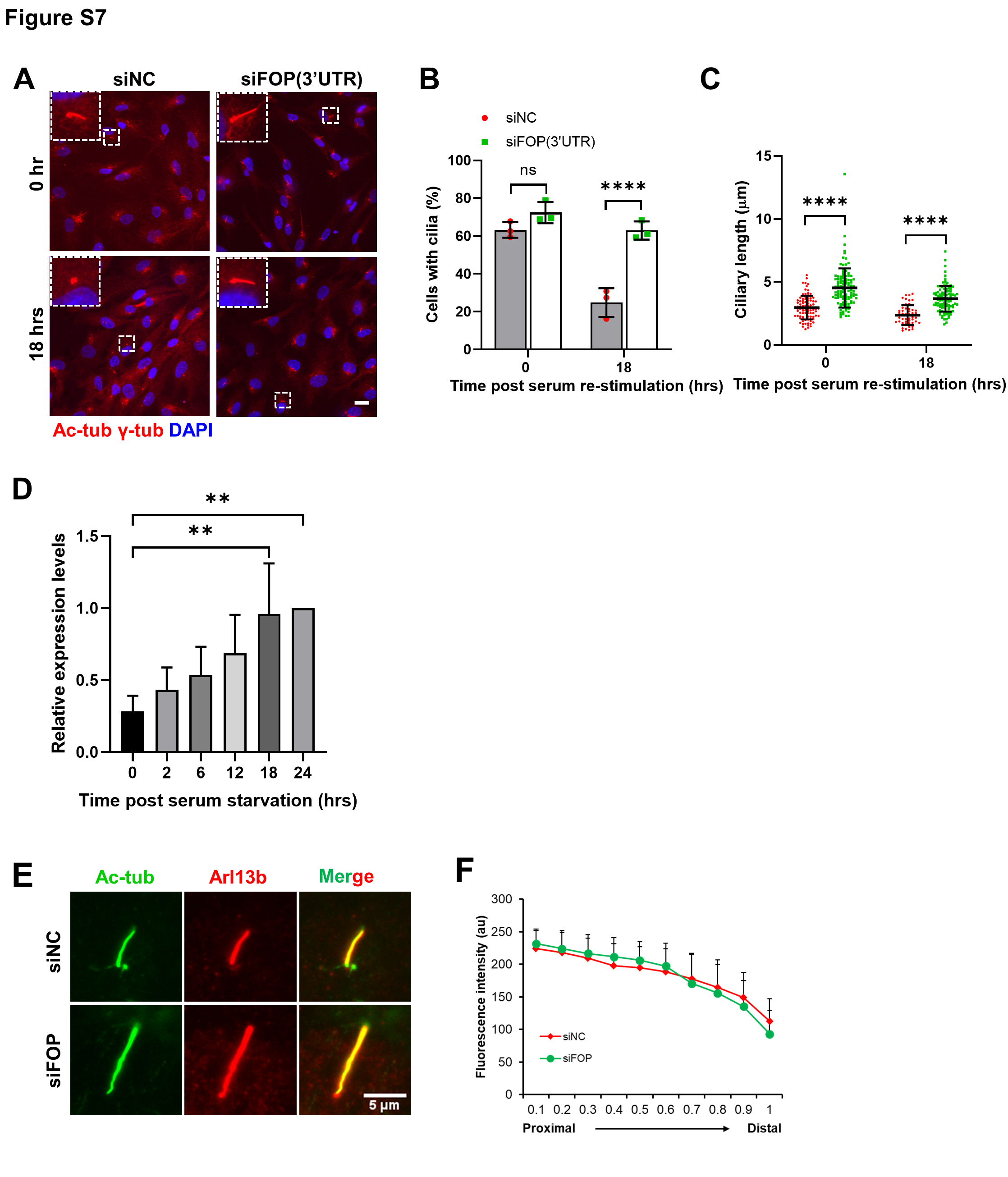

Supplement: Supplementary file 8 [file Image_7.TIF]

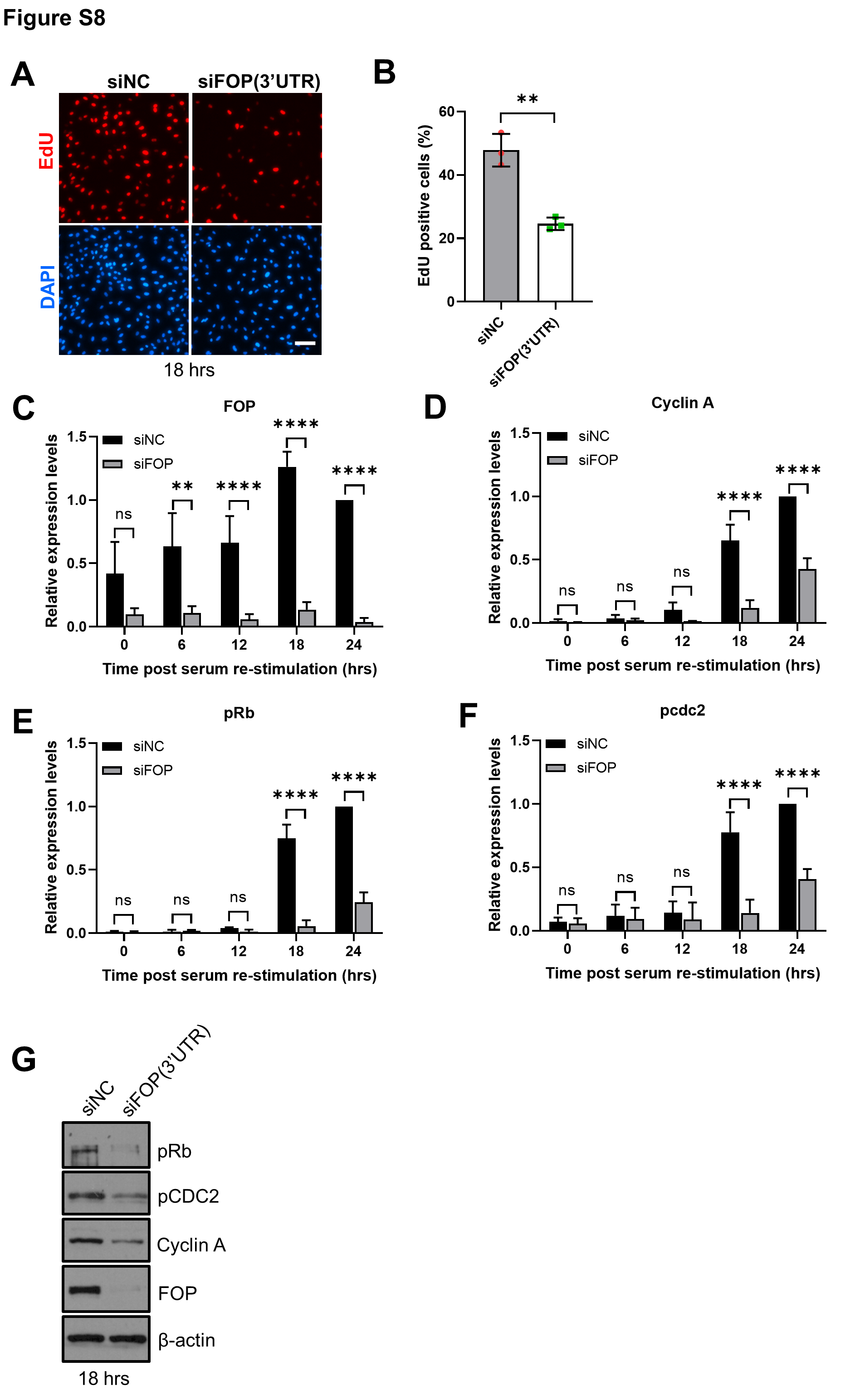

Supplement: Supplementary file 9 [file Image_8.TIF]

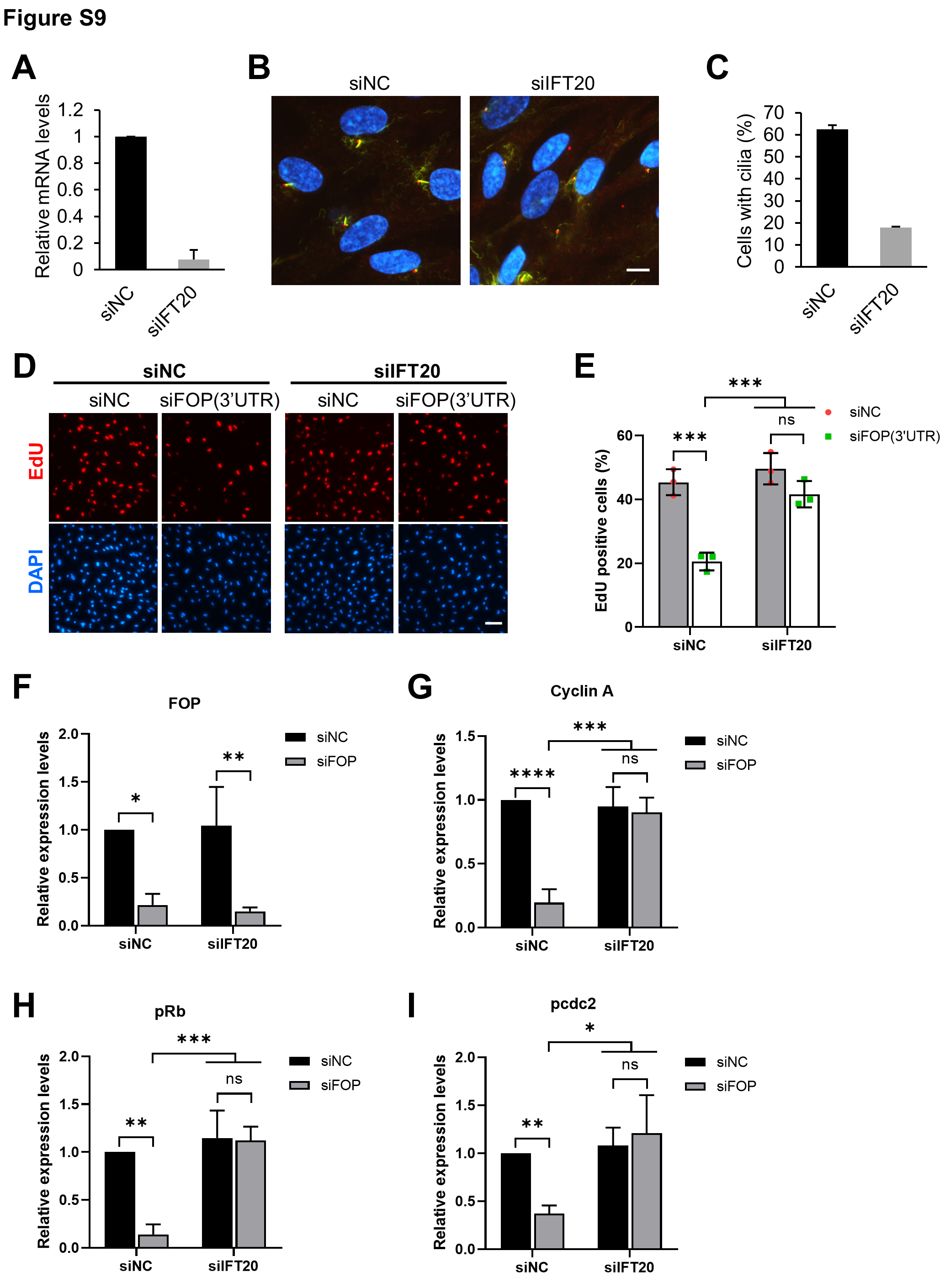

Supplement: Supplementary file 10 [file Image_9.TIF]
